# Hsa_circ_0001756 drives gastric cancer glycolysis by increasing the expression and stability of PGK1 mRNA

**DOI:** 10.3389/fimmu.2025.1511247

**Published:** 2025-02-20

**Authors:** Long Qian, Luman Wang, Hao Chen, Song Wang, Yinfen Hou, Li Xu, Yabin Xia, Maoqi Xu, Xiaoxu Huang

**Affiliations:** ^1^ Department of Gastrointestinal Surgery, The First Affiliated Yijishan Hospital of Wannan Medical College, Wuhu, Anhui, China; ^2^ General Surgery Department, Wuhu Hospital of Traditional Chinese Medicine, Wuhu, Anhui, China; ^3^ Anhui Province Key Laboratory of Non-coding RNA Basic and Clinical Transformation, Wuhu, Anhui, China; ^4^ Department of Immunology, School of Basic Medical Sciences, Fudan University, Shanghai, China; ^5^ Department of Oncology, Wuhu Conch Hospital, Wuhu, Anhui, China; ^6^ Department of Medical Examination Center, The First Affiliated Yijishan Hospital of Wannan Medical College, Wuhu, Anhui, China; ^7^ General Surgery Department, Anhui Wannan Rehabilitation Hospital, The Fifth People’s Hospital of Wuhu, Wuhu, Anhui, China

**Keywords:** circRNA, glycolysis, miR-185-3p, PGK1, PTBP1

## Abstract

**Introduction:**

Strategies for preventing high glycolysis in tumour cells are urgently needed. CircRNAs (circRNAs) play important roles in glycolysis. However, the mechanism underlying the effects of hsa_circ_0001756 in gastric cancer (GC) remains unclear.

**Methods:**

In this study, we detected the expression of hsa_circ_0001756 in GC tissues and cells using quantitative real-time polymerase chain reaction (qRT PCR). Construct a silencing and overexpression vector to validate the role of hsa_circ_0001756 in GC. Pulldown and RIP experiments were conducted to verify the identification of miRNA and protein binding to hsa_circ_0001756.

**Results:**

The expression level of hsa_circ_0001756 in GC tissues and cells is significantly upregulated. The expression level of hsa_circ_0001756 is closely related to TNM stage and tumour size in patients with GC. The proliferation and migration of hsa_circ_0001756-expressing cells *in vitro* were assessed by functional experiments. Hsa_circ_0001756 was found to not only promote the expression and stability of PGK1 by binding with polypyrimidine tract-binding protein 1 (PTBP1) but also promote glycolysis through the miR-185-3P/PGK1 pathway. We found that the regulatory relationships of competing endogenous RNA (ceRNA) and RNA-binding proteins (RBPs) with hsa_circ_0001756may affect glycolysis in GC.

**Conclusion:**

This study provides a theoretical basis for designing drugs that target molecules related to energy metabolism in tumours and provides a new strategy for the clinical treatment of GC.

## 1 Background

Early diagnosis of gastric cancer (GC) is difficult. Therefore, the 5-year survival rate of patients with GC is very low (1). Systemic chemotherapy and palliative surgery for advanced GC have poor overall outcomes, and the median survival is only 8–11 months (2). In recent years, molecular targeted therapies for GC, which embody the concept of precision medicine, have gradually entered the clinic and have received widespread attention. However, the curative effects of GC-targeted drugs currently in clinical use are still unclear. Finding biomarkers and molecular targets for GC is very important. In the 1930s, Warburg discovered that the level of glycolysis in tumor cells was markedly greater than that in normal cells and named this phenomenon the Warburg effect (3). Studies have shown that glycolysis is the main energy source in tumor cells and maintains the growth of these cells (4). Recent studies have shown that inhibiting glycolysis significantly reduces the proliferation of GC cells (5, 6). These results indicate that glycolysis is a key factor affecting the proliferation of GC cells. Phosphoglycerate kinase 1 (PGK1) is a key enzyme in glycolysis and plays an important role in cellular energy metabolism. Exploration of the key factors that regulate glycolysis in GC should reveal potential targets for controlling GC cell growth and proliferation.

Circular RNAs (circRNAs) can function in various ways and often act as microRNAs (miRNAs) to influence cell proliferation (7) and apoptosis (8) in various biological processes. CircRNAs are covalently closed circular RNA molecules formed by “backsplicing” of pre-mRNA transcripts and are thus stable molecules (9). Many previous studies have demonstrated that circRNAs can promote or inhibit the development of various tumors. For example, circAKT3 enhances GC resistance to cisplatin via the miR-198/PIK3R1 axis (10). Hsa_circ_0001756 is a recently popular non coding RNA, which is a metabolism related circRNA that can promote the proliferation of various tumor cells (11, 12). We will use this as a cornerstone to focus our attention on the yet to be fully explored tumor type Hsa_circ_0001756. And through organizational and cytological verification, Hsa_circ_0001756 has differential expression in gastric cancer, further analyzing its regulatory mechanisms in different processes such as occurrence, development, metastasis, and glycolysis in gastric cancer. And it is expected that in new tumor research, Hsa_circ_0001756 will be used as a new therapeutic target to explore the specific regulatory mechanism of glycolysis in gastric cancer, providing new ideas and directions for finding potential therapeutic targets. As small noncoding RNAs, miRNAs act on the 3’ untranslated regions (UTRs) of mRNAs, thus directly regulating their expression; affecting cell proliferation, differentiation and other functions; and indirectly affecting various physiological and pathological processes in the body (13). According to previous reports, miRNA-185-3p is involved in various tumors and has important functions (14, 15). RNA-binding proteins (RBPs) also play important roles in the function of competing endogenous RNAs (circRNAs) (16). As one of the key enzymes in the glycolytic pathway, PGK1 is involved in many biological activities. Increased expression of PGK1 in cells can promote the occurrence of tumors (17, 18), and PGK1 expression is regulated by a variety of miRNAs (19).

In this study, we explored whether hsa_circ_0001756 has a regulatory role in GC. Here, we first verified that hsa_circ_0001756 is differentially expressed in GC. Moreover, GC cell proliferation was measured *in vitro* and *in vivo* after transfection of interference and overexpression vectors to further explore the mechanism of action of hsa_circ_0001756. The goal of this study was to identify new molecular targets for GC and new strategies for treating the disease.

## 2 Materials and methods

### 2.1 Clinical data

Seventy-four patients who underwent GC surgery at Yijishan Hospital, the First Affiliated Hospital of Wannan Medical College (Wuhu, China), from 2015–2019 were enrolled. This study was approved by the ethics committee of the institution. During surgery, GC tissue was collected in a timely manner in a tube, and paired paracarcinoma tissue was collected from 5 cm above the edge of the GC tissue. Regular follow-ups were conducted. Patient details are listed in **Supplementary Table S7**.

### 2.2 Cell culture

Four GC cell lines (MKN-45, HGC-27, MGC-803, and AGS) and normal human gastric mucosa epithelial (GES-1) cells were purchased from the Cell Bank of the Chinese Academy of Sciences (Shanghai, China). The cells were cultured in RPMI 1640 medium (Beyotime, China) containing 10% fetal bovine serum and the antibiotics penicillin and streptomycin (Beyotime, China) at 37°C in a cell incubator containing 5% CO2.

### 2.3 RNA extraction, genomic DNA extraction and qRT–PCR

Total RNA was extracted from cells, paracarcinoma tissues, and GC tissues using TRIzol (Beyotime, China) according to the manufacturer’s instructions. cDNA was synthesized with a reverse transcription reagent (Beyotime, China), and qRT−PCR was performed using cDNA as a template. The reaction conditions were 95°C for 2 min, 95°C for 15 s, 60°C for 1 min, and 72°C for 30 s (40 cycles). GAPDH was used as the internal reference gene for circRNA_0001756, and U6 was used as the internal reference gene for miRNA-185-3p; relative expression was calculated by the 2-ΔΔCt method. The primer sequences are shown in **Supplementary Table S1**.

### 2.4 miRNA, short interfering RNA and plasmid construction

siRNAs and miRNA inhibitors and mimics were synthesized by GenePharma (Shanghai, China). An hsa_circ_0001756 overexpression plasmid was synthesized by Shanghai Hanheng Company. The sequences are shown in **Supplementary Tables S2**, **S3**. One night prior to the experiment, normal cultured cells were digested and spread on six-well plates at a density of 200,000 cells per well. On the second day, the culture medium was removed, and the cells were washed with PBS. Lipo2000 was mixed with 50 µmol siRNA followed by 50 µL of serum-free or antibiotic-free culture medium. Then, 2 µL of Lipo2000 was added to a new EP tube, 50 µL of culture solution was added, and the mixture was gently mixed. The solutions in the two tubes were then mixed and allowed to stand at room temperature for 20 min. Next, 2 mL of medium was added to each well of a 6-well plate, the mixture was added to each well, and the plate was shaken well. The 6-well plate was then cultured in an incubator. After 6–8 h, the transfection medium was discarded, and culture medium containing serum without antibiotics was added. The cells were further cultured, and cell experiments were performed 48 h after transfection.

### 2.5 RNase R treatment

Two micrograms of RNA was digested with RNase R (Beyotime, China) for 5 min, and qRT−PCR was used to measure hsa_circ_0001756 and linear PGK1 levels.

### 2.6 ActD treatment

Different GC cells were treated with 2 μg/mL ActD (Sigma minus;Aldrich, USA) for a period, and qRT−PCR was performed to measure hsa_circ_0001756 and linear PGK1 levels.

### 2.7 Western blotting

Four hundred microliters of RIPA lysis buffer was added to each group of GC cells to extract total protein, and the samples were separated by SDS−PAGE and then transferred to a nitrocellulose (NC) membrane. Rapid blocking solution was used for blocking (Beyotime, China) for 20 min. The membrane was incubated overnight with diluted primary antibodies [against PGK1 (1:1000, ABclonal), HK2 (1:1000, ABclonal), GLUT1 (1:1000, ABclonal), β-actin (1:4000, ABclonal) and PTBP1 (1:1500, ABclonal)] and then with diluted secondary antibodies (1:2000) at 37°C for 2 h and washed with TBST. ECL reagent was added dropwise, and the membrane was exposed in a darkroom. The grey values of each protein band were analyzed by ImageJ software.

### 2.8 Immunohistochemistry

Transplanted tumor tissues were embedded in paraffin, dewaxed, hydrated with xylene and graded alcohol solutions, and washed for 3 min with PBS. A primary antibody against PGK1 (1:1000, ABclonal, China) or Ki67 (1:1000, Abcam, USA); BAX (1:1000, Abcam, USA) was added, and the tumor tissues were refrigerated overnight. Secondary antibody was added, and the tumor tissues were incubated at room temperature for 30 min. Fresh diaminobenzidine (DAB) and hematoxylin solution were added for staining. After dehydration, photographs were taken under a microscope at 400×.

### 2.9 RNA fluorescence *in situ* hybridization

hsa_circ_0001756 and miR-185-3p probes were synthesized by RiboBio. The probe were detected with a FISH kit (RiboBio). Immunofluorescence images were taken with a Zeiss laser scanning microscope. The FISH probes used are shown in **Supplementary Table S4**.

### 2.10 Immunofluorescence assay

GC cells (3 × 10^4^) were plated in confocal dishes one night prior to the experiment. On the day of the experiment, the cells were fixed, permeabilized, and blocked with 2% BSA for 2 h at room temperature. The cells were incubated with primary antibody overnight, with a fluorescent secondary antibody for 1 h, and then with DAPI for 10 min. An LSM 710 confocal microscope (Zeiss, Germany) was used for image acquisition.

### 2.11 RNA pull-down assay

HGC-27 GC cells were transfected and cultured for 48 h and collected for the isolation of total protein. Each circRNA probe and NC probe (100 pmol) were denatured at 95°C for 3 min and then immediately placed on ice for denaturation and to maintain the linear structure of the probe. One milligram of total protein was placed into two 1.5-mL EP tubes, the denatured circRNA probe and NC probe were added, and the total protein-probe mixture was incubated overnight in RIP buffer at 4°C with vertical mixing. The next day, 60 µL of prewashed streptavidin magnetic beads (Dynabeads™ Streptavidin Trial Kit, Invitrogen) were added to capture the RNA−protein complexes. The magnetic beads were collected on a magnetic stand, washed with 1 mL of RIP buffer, and subjected to five 5-min washes with vertical mixing. The magnetic beads were then collected, 30 µL of SDS loading buffer was added to resuspend the magnetic beads, and the proteins captured by RNA pull-down were eluted at 95°C for 5 min. Ten microliters of the above samples were separated by 8%~12% SDS−PAGE, high-resolution silver staining was performed, and the unique bands in each sample were isolated for mass spectrometry analysis. The sequences of the probes are shown in **Supplementary Table S5**.

### 2.12 RBP immunoprecipitation

The hsa_circ_0001756 overexpression vector was cotransfected with a GFP fusion protein. An anti-GFP antibody and an RNA immunoprecipitation kit were used according to the kit instructions, and the magnetic beads were cleaned with RNA lysis solution to extract RNA. The expression of miR-185-3p was measured by PCR with specific primers. The regulatory effect of miR-185-3p on PGK1 expression was verified using a previously described method. The sequences of the biotinylated probes are listed in **Supplementary File 1**, **Suppplementary Table S6**.

### 2.13 Measurement of luciferase reporter activity

Various plasmids were used for the synthesis of a dual luciferase reporter system (Nanjing Gene Regulation Company); mutant and control miR-185-3p plasmids were transfected into hsa_circ_0001756-overexpressing, hsa_circ_0001756 knockdown and corresponding control cell lines. Forty-eight hours after transfection, the fluorescence intensity was measured by a fluorescence detector; Renilla luciferase activity was used as an internal reference. The regulatory effect of miR-185-3p on PGK1 expression was verified using a previously described method.

### 2.14 *In vitro* functional experiments

The methods used for flow cytometry analysis of apoptosis, the cell proliferation assay, the scratch healing test, migration and invasion analyses, cell cloning experiments (20), and measurement of glycolytic function can be found in the **Supplementary Methods**.

### 2.15 *In vivo* experiments on nude mice

Nude mice were purchased from Qinglongshan Animal Factory, Nanjing, China, and 1 × 10^6^ lentivirus-transfected GC cells were subcutaneously administered. The animals were injected once a week and sacrificed after 21 days. The tumor size was observed at intervals of 3 days, and the tumors were weighed at the time of sacrifice. The ethics committee approved the experiments, and the protocols were performed in accordance with the relevant regulations of the Animal Protection Association.

### 2.16 Statistical analysis

The data were analyzed with GraphPad Prism V8.0 software (La Jolla, CA, USA). Kaplan−Meier analysis was used to generate long-term survival curves. The data are presented as the (X ± S) of three independent experiments. Linear regression analysis was used to analyze the correlation between hsa_circ_0001756 and miR-185-3P expression in GC samples. Data were compared between two groups by independent sample t tests, and data were compared among multiple groups by one-way ANOVA. Statistical significance was defined as P<0.05.

## 3 Results

### 3.1 Hsa_circ_0001756 is overexpressed in GC tissues

**Figure 1:**
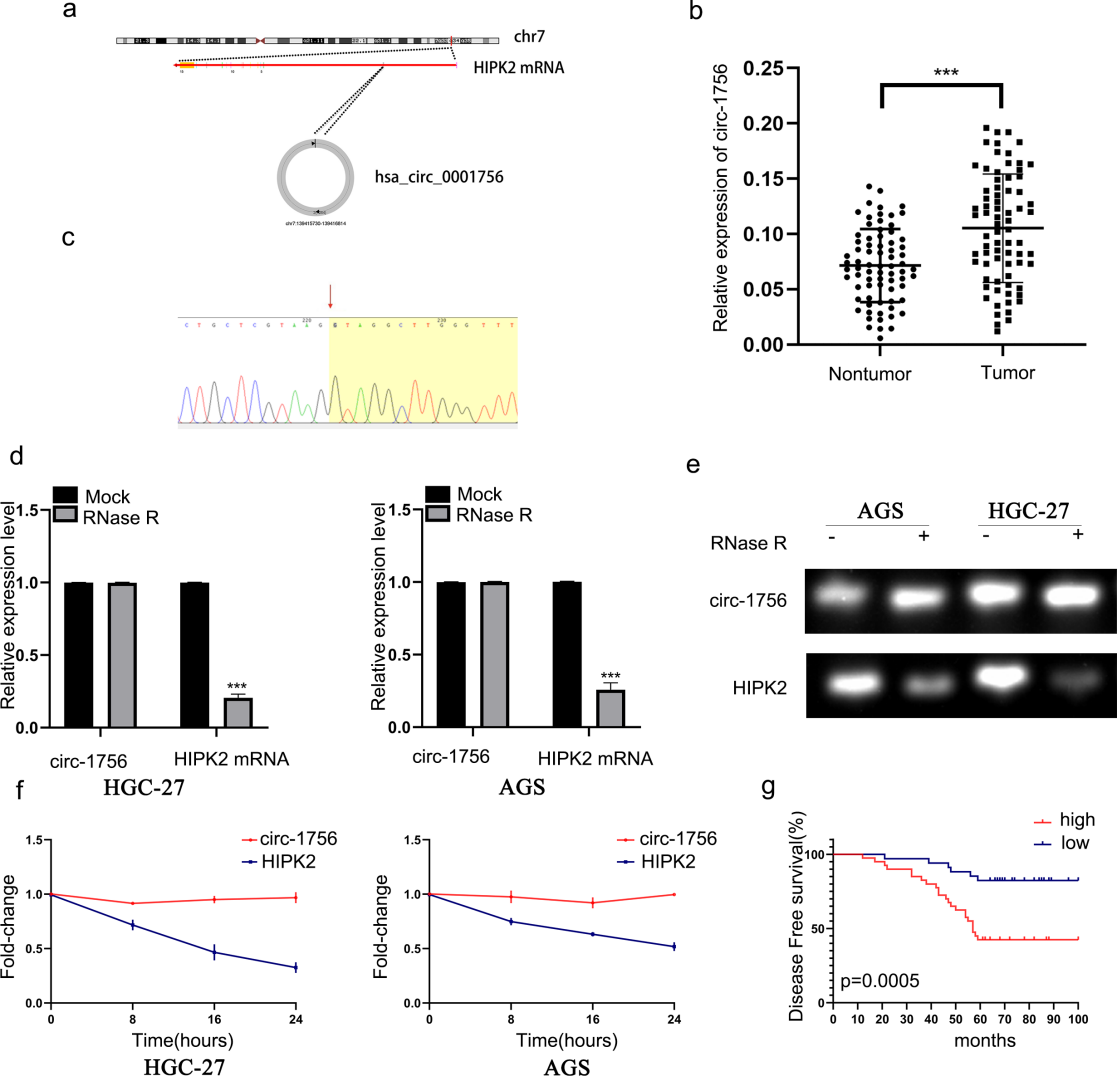
Hsa_circ_0001756 is overexpressed in GC tissues. **(a)** The schematic diagram shows that hsa_circ_0001756 (circ_1756) is formed by the loops of exon 2 of the HIPK2 gene, located on chromosome 7. **(b)** qRT–PCR analysis of seventy-four pairs of GC and adjacent nontumor tissues was performed to measure the expression of hsa_circ_0001756 (n = 74, p < 0.0001, Student’s t tests). **(c)** The PCR product of hsa_circ_0001756 was sequenced by Sanger sequencing. The red arrow indicates the splicing site. **(d, e)** qRT−PCR and RT−PCR results showing linear HIPK2 and hsa_circ_0001756 expression levels under RNase R treatment. **(f)** qRT–PCR analysis of the mRNA expression of circ-1756 and HIPK2 in GC cell lines after treatment with ActD. **(g)** Association between hsa_circ_0001756 expression levels and DFS according to Kaplan–Meier survival curve analysis. The data are shown as the means ± SDs; Linear regression analysis was used to analyze the correlation in b; Student’s t‐test and ANOVA compared the difference in d, f, Log‐rank test for survival comparison in **(g)** ***p<0.001.

Hsa_circ_0001756 is formed by a loop of exon 2 of the HIPK2 gene, located on chromosome 7, and is 1084 bases long (**Figure 1A**). In 74 pairs of GC and adjacent nontumor tissues, hsa_circ_0001756 was overexpressed (P<0.0001) (**Figure 1B**). Patient data are shown in **Supplementary Table S7**. Then, we performed statistical analysis. Increased hsa_circ_0001756 expression was correlated with tumor size and T and N stage (**Table 1**). Sanger sequencing of the PCR products revealed that the circular structure of the circRNA was formed by head-to-tail splicing (**Figure 1C**). One of the key characteristics of circRNAs is their stability (21–23). AGS and HGC-27 cells were digested with the RNase R enzyme, and the qRT−PCR results revealed that the expression of linear HIPK2 decreased, while there was little change in hsa_circ_0001756 expression (**Figures 1D, E**). The mRNA level of linear HIPK2 gradually decreased over time, whereas the level of hsa_circ_0001756 was more stable and not affected by ActD treatment (**Figure 1F**). In addition, we performed long-term postoperative follow-up. A total of 74 GC patients were divided into circ_0001756^low^ and circ_0001756^high^ groups according to the median expression level. Statistical analysis indicated that the prognosis of patients with high expression of hsa_circ_00017565 was worse than that of patients with low expression of hsa_circ_00017565 (**Figure 1G**).

**Table 1:** Correlations between the expression of hsa_circ_0001756 and various clinicopathological features in 74 GC patients.

Characteristics		hsa_circ_0001756 expression		p value
		low	high	
All cases	74	34	40	
Age (years)				
≤ 60	34	19	15	0.1138
> 60	40	15	25	
Gender				
Male	37	19	18	0.7392
Female	37	15	22	
Tumor size (cm)				
≤ 5	32	20	12	0.012*
> 5	42	14	28	
T stage				
T1-T2	25	16	9	0.026*
T3-T4	49	18	31	
N stage				
N0-N1	34	21	13	0.0118*
N2-N3	40	13	27	
M stage				
M0	58	27	31	0.8422
M1	16	7	9	

*p<0.05.

### 3.2 Hsa_circ_0001756 promotes glycolysis, proliferation, migration and migration in GC

**Figure 2:**
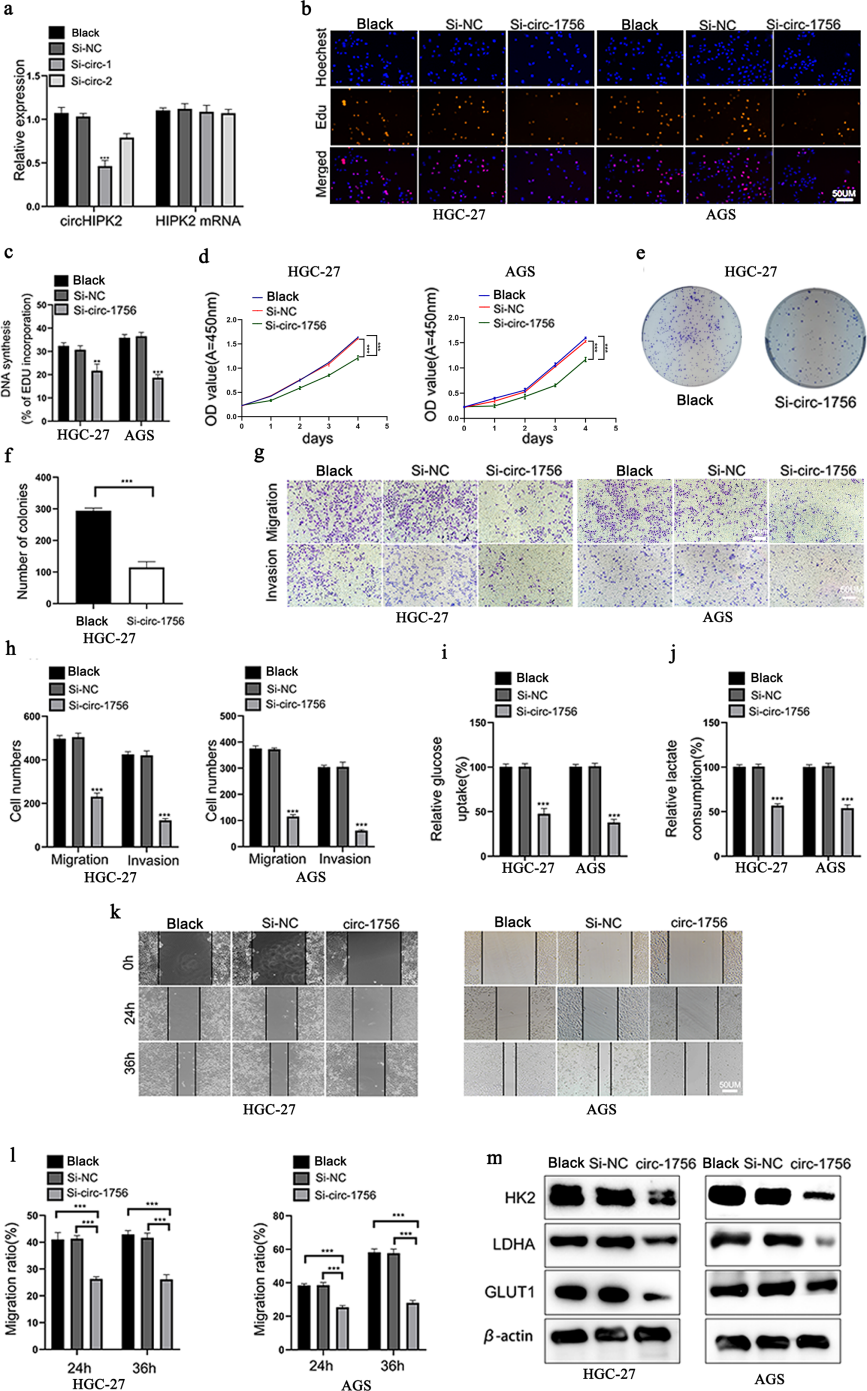
Hsa_circ_0001756 promotes glycolysis, proliferation, and migration in GC. **(a)** The mRNA expression levels of hsa_circ_0001756 and HIPK2 in cells treated with or without siRNA. **(b)** Analysis of HGC-27 and AGS cell proliferation after transfection with hsa_circ_0001756 siRNA (si-circ-1756) or control siRNA (Si-NC) by EdU staining. Scale bar: 50 μm. **(c)** Statistical analysis of the proportion of EdU-positive cells relative to transfected GC cells. **(d)** Analysis of HGC-27 and AGS cell proliferation after transfection of Si_circ_1756 or Si-NC by the CCK-8 assay. **(e)** Analysis of cell proliferation and colony formation after Si_circ_1756 or Si-NC transfection into HGC-27 cells. **(f)** Statistical analysis of the colony formation rate of transfected GC cells. **(g)** Assessment of the invasion and migration of hsa_circ_0001756-silenced (Si-circ-1756) and control (Si-NC) HGC-27 and AGS cells by the Transwell assay. Scale bar: 50 μm. **(h)** Statistical analysis of the number of transfected AGS and HGC-27 cells that passed through the Transwell membrane. **(i)** Relative glucose uptake by transfected AGS and HGC-27 cells was measured. **(j)** Relative lactate production by transfected AGS and HGC-27 cells was measured. **(k)** Wound healing in the transfected AGS and HGC-27 cell groups. Scale bar: 50 μm. **(l)** Statistical analysis of cell migration in the wound healing assay. **(m)** The protein levels of HK2, LDHA and GLUT1 in transfected AGS and HGC-27 cells were analyzed by Western blotting. The data are expressed as the means ± SDs of three experiments. Student’s t‐test and ANOVA analyzed the difference in **(a, c, d, f, h–j, l)**. The data are shown as the means ± SDs; **p<0.01, ***p<0.001.

To explore the role of hsa_circ_0001756 in GC cells, We measured hsa_circ_0001756 expression levels in different GC cells. AGS cells were found to have the highest hsa_circ_0001756 expression (**Supplementary Figure S1A**).we designed siRNA oligonucleotides (Si-circ-1 and Si-circ-2). Si-circ-1 successfully reduced hsa_circ_0001756 expression, but the transcript levels of endogenous linear HIPK2 in HGC-27 and AGS cells were not affected (**Figure 2A**). We subsequently performed a series of functional experiments in which Si-circ-1 (si-circ_1756) was used to knockdown hsa_circ_0001756. EdU staining, the CCK-8 assay and the cell colony formation assay revealed that silencing hsa_circ_0001756 decreased the proliferative ability of GC cells (**Figures 2B–F**) (**Supplementary Figure S2B**). In addition, Transwell assays (**Figures 2G, H**) revealed reduced migration and invasion of GC cells in which hsa_circ_0001756 was silenced. Subsequent analysis of the Warburg effect revealed that hsa_circ_0001756 knockdown reduced glucose uptake and lactate production (**Figures 2I, J**). The scratch test detected the migration ability of gastric cancer cells,(**Figures 2K, L**). HK2 (24), LDHA (25) and GLUT1 (26) are the key enzymes involved in glycolysis. Silencing circ_0001756 reduced the expression of these glucose-metabolizing enzymes(**Figure 2M**). Therefore, we conclude that circ_0001756 silencing inhibits proliferation, migration, invasion and glycolysis.

We further explored the effect of hsa_circ_0001756 overexpression. qRT−PCR was used to assess the transfection efficiency in the overexpression experiments (**Supplementary Figure S2A**). EdU staining and the CCK-8 assay confirmed that overexpression of hsa_circ_0001756 increased the proliferation of GC cells (**Supplementary Figures S2B–D**), and the scratch test (**Supplementary Figures S2E, F**) revealed that overexpression of hsa_circ_0001756 increased the migration of GC cells.

### 3.3 Hsa_circ_0001756 acts as a molecular sponge of miRNA-185-3p

**Figure 3:**
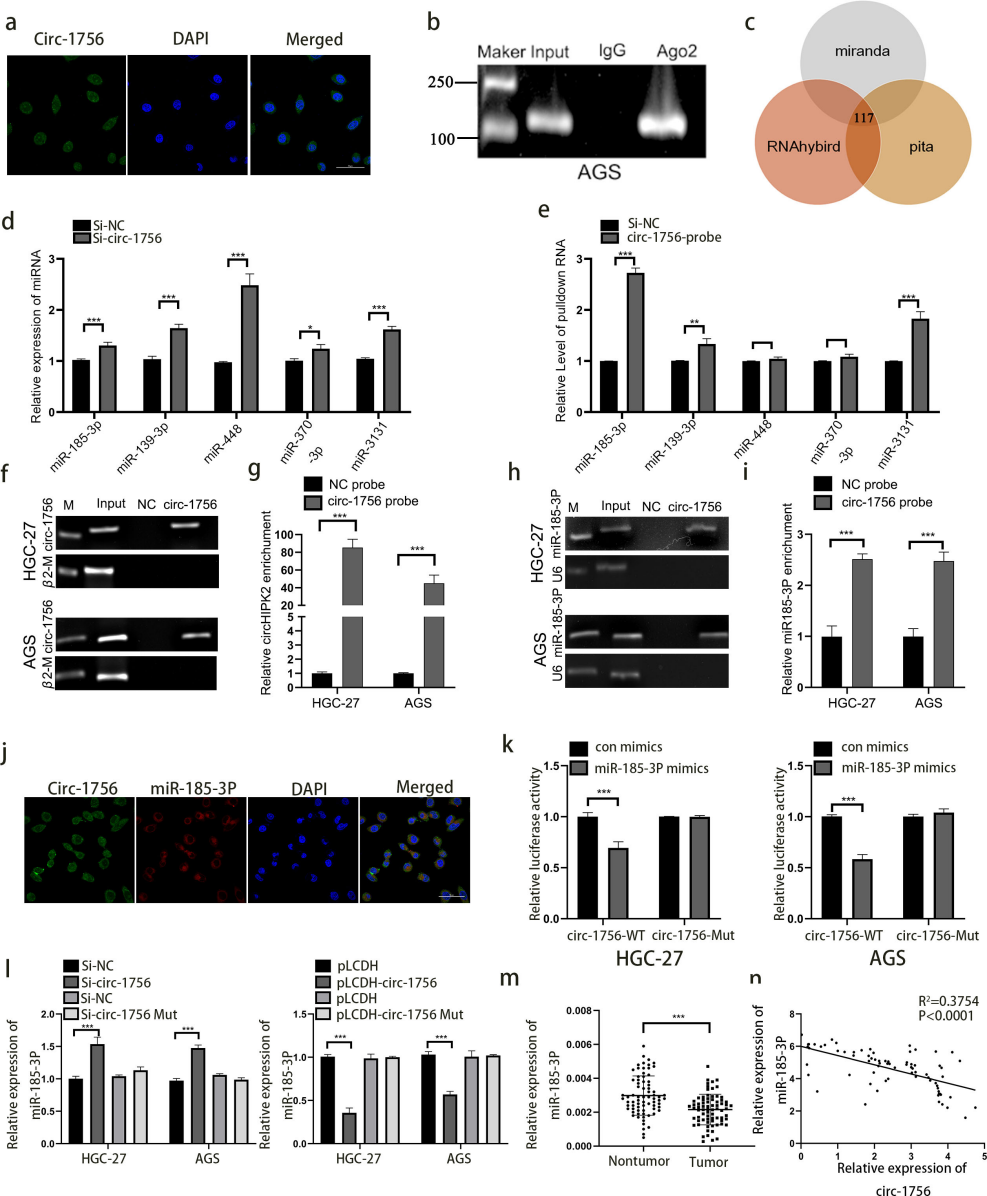
Hsa_circ_0001756 acts as a molecular sponge of miRNA-185-3p. **(a)** FISH was used to determine the subcellular localization of hsa_circ_0001756 (circ_1756) in GC cells. **(b)** RIP analysis of hsa_circ_0001756 levels in immunoprecipitates obtained with an anti-AGO2 antibody from GC cells. **(c)** Schematic diagram showing the overlapping miRNAs with the potential to bind hsa_circ_0001756 predicated by different databases. **(d)** qPCR was performed to evaluate the expression levels of five predicted target miRNAs in hsa_circ_0001756-silenced HGC-27 cells. **(e)** miRNAs were pulled down by a biotin-labelled probe targeting either random sequences or the hsa_circ_0001756 black-splice junction sequence. The enrichment of the five predicted miRNAs was analyzed via qPCR. **(f–i)** Lysates prepared from AGS cells and HGC-27 cells treated with hsa_circ_0001756 were incubated with a biotinylated probe targeting hsa_circ_0001756, after which an RNA pull-down assay was performed. qRT–PCR and RT–PCR were used to determine hsa_circ_0001756 **(f, g)** and miR-185-3p expression **(h, i)**. **(j)** FISH was used to determine the subcellular localization of hsa_circ_0001756 (circ_1756) and miR-185-3p in GC cells. **(k)** AGS and HGC-27 cells were cotransfected with WT or Mut reporter gene constructs and control or miR-185-3p mimics, and 48 hours after transfection, dual-luciferase reporter activity was determined. **(l)** qRT–PCR was used to determine miR-185-3p expression in AGS and HGC-27 cells transfected with hsa_circ_0001756 siRNA (Si-circ-1756) or mutant hsa_circ_0001756 siRNA (Si-circ-1756-mut). **(m)** qRT–PCR analysis of miR-185-3p expression in GC tissues and adjacent tissues. Paired t test (n  = 74). **(n)** Regression analysis revealed that miR-185-3p and hsa_circ_0001756 levels were negatively correlated (n  = 74). The data are shown as the means ± SDs; Student’s t‐test and ANOVA analyzed the difference IN **(d, e)**. **(g, i, k, l)** Linear regression analysis was used to analyze the correlation in **(m, n)**. *p<0.05, **p<0.01, ***p<0.001.

Next, we explored whether hsa_circ_0001756 has other effects. CircRNAs have different functions in different subcellular compartments (27). According to FISH and Subcellular Separation Experiment, hsa_circ_0001756 was mainly localized in the cytoplasm (**Figure 3A**) (**Supplementary Figure S1C**). According to previous reports, cytoplasmic circRNAs can often bind miRNAs (28). Therefore, RIP assay were performed to verify the ability of hsa_circ_0001756 to bind to miRNAs in GC cells. We used an anti-AGO2 antibody to pull down and amplify hsa_circ_0001756 and found that hsa_circ_0001756 acted as a molecular sponge and bound AGO2 miRNA (**Figure 3B**) (**Supplementary Figure S1D**).

We predicted the miRNAs that might bind hsa_circ_0001756 (**Supplementary Table S10**) through various databases (miRanda, RNAhybird and pita) and identified 117 overlapping miRNAs predicted by all three databases. According to previous reports, 5 of these miRNAs have carcinogenic functions (hsa-miR-185-3p, hsa-miR-139-3p, hsa-miR-448, hsa-miR-370-3p, and hsa-miR-3131) (**Figure 3C**). We then knocked down hsa_circ_0001756 to observe the changes in the expression of these miRNAs (**Figure 3D**). A pull-down assay combined with qPCR revealed that hsa_circ_0001756 could specifically bind to these miRNAs, especially miR-185-3p, and miR-448 and miR-370-3p cannot be significantly enriched. (P<0.001) (**Figure 3E**). RNAhybrid prediction analysis revealed that hsa_circ_0001756 and miR-185-3p have 8 stable binding regions [maximum binding free energy (MFE)>-20 kcal/mol] (**Supplementary Figure S4A**). In summary, miR-185-3p is the most likely miRNA to bind hsa_circ_0001756. Therefore, we performed a pull-down assay with a biotin-conjugated hsa_circ_0001756 probe to determine whether hsa_circ_0001756 can interact with miR-185-3p. The results revealed that miR-185-3p levels were obviously increased in the biotin-conjugated hsa_circ_0001756 probe group compared with the negative control group, suggesting that hsa_circ_0001756 could directly interact with miR-185-3p (**Figures 3F–I**). FISH revealed that both hsa_circ_0001756 and miR-185-3p were localized mainly in the cytoplasm (**Figure 3J**). In addition, miR-185-3p level increased after hsa_circ_0001756 was silenced and decreased after hsa_circ_0001756 was overexpressed. However, miR-185-3p expression was almost unchanged in the groups in which hsa_circ_0001756 with mutations in the miR-185-3p binding site mutant was silenced or overexpressed (**Figure 3L**). A dual-luciferase reporter assay revealed that miR-185-3p bound to hsa_circ_0001756-WT (circ-1756-WT) (**Supplementary Figure S3A**) (**Figure 3K**). In addition, we analyzed miR-185-3p expression in GC tissue by qRT−PCR and found that it was decreased in tumors (**Figure 3M**), and statistical analysis revealed that hsa_circ_0001756 expression was negatively correlated with miR-185-3p expression (P<0.0001) (**Figure 3N**). In summary, hsa_circ_0001756 can competitively bind to miR-185-3p to exert its function in GC.

### 3.4 Inhibition of miR-185-3p increases glycolysis through PGK1

After elucidating the relationship between hsa_circ_0001756 and miR-185-3p, we next studied how miR-185-3p plays a role in GC. qRT−PCR was used to verify the transfection efficiency of miR-185-3p mimics (Additional file 7, **Supplementary Figure S6A**). EdU staining, the colony formation assay and the CCK-8 assay revealed that the miR-185-3p mimics inhibited GC cell proliferation (Additional file 7, **Supplementary Figures S6B–D, I, J**). Moreover, the miR-185-3p mimics inhibited cell invasion and migration (Additional file 7, **Supplementary Figures S6E–H**). The miRNA mimics also inhibited glycolytic function and decreased glucose uptake and lactate production (Additional file 7, **Supplementary Figures S6L, M**) and inhibited the protein expression of HK2, LDHA, and GLUT1 (Additional file 7, **Supplementary Figure S6K**).

To clarify the mechanism underlying the effect of miR-185-3p, we used the TargetScan database to determine that miR-185-3p can bind to the 3’-UTR of PGK1 (Additional file 5, **Supplementary Figure S4B**). This finding indicates that PGK1 may be a target of miR-185-3p. After miR-185-3p overexpression, the expression of PGK1 was significantly decreased, while the protein level of PGK1 was increased by the inhibition of miR-185-3p (Additional file 7, **Supplementary Figure S6P**). The miR-185-3p mimic significantly decreased PGK1 mRNA levels, whereas the miR-185-3p inhibitor increased PGK1 mRNA levels (Additional file 7 **Supplementary Figure S6N**).

We investigated whether miR-185-3p exerts tumor-suppressive effects through PGK1. We found that miR-185-3p targeted the 3’-UTR of PGK1 (**Supplementary Figure S3B**); in addition, a dual-luciferase reporter assay revealed that miR-185-3p bound to PGK1-WT (Additional file 7, **Supplementary Figure S6O**).

We observed the effect of hsa_circ_0001756 on PGK1 expression, and Western blot analysis revealed that the protein expression of PGK1 decreased after hsa_circ_0001756 was silenced (Additional file 7 **Supplementary Figure S6Q**). we analyzed PGK1 expression in GC tissue by qRT−PCR(Additional file 7 **Supplementary Figure S6R**). PGK1 is positively correlated with circHIPK2., miR185-3p is negatively correlated with the expression level of PGK1(Additional file 7 **Supplementary Figures S6S, T**).

### 3.5 Downregulation of miR-185-3p expression reverses the effect of hsa_circ_0001756 silencing in GC cells

To determine whether hsa_circ_0001756 exerts a tumor-promoting effect through miR-185-3p, we conducted a response experiment. We cotransfected an hsa_circ_0001756 siRNA (Si-circ_0001756) and the miR-185-3p inhibitor into GC cells and then observed whether the tumor-suppressive effect of silencing hsa_circ_0001756 was affected by the miR-185-3p inhibitor. The results showed that the miR-185-3p inhibitor could restore the inhibitory effect of hsa_circ_0001756 silencing on GC cells, affecting glucose consumption (**Supplementary Figure S5A**), lactate production (**Supplementary Figure S5B**), proliferative capacity (Additional file 8, **Supplementary Figures S7A–C, F, G**), behavior in the scratch test (Additional file 8, **Supplementary Figures S7D, E**) and Transwell assay (Additional file 8, **Supplementary Figures S7H, I**).

We next examined whether a miR-185-3p inhibitor could attenuate the downregulation of PGK1 expression induced by hsa_circ_0001756. The hsa_circ_0001756-mediated downregulation of PGK1 expression was reversed by the miR-185-3p inhibitor (Additional file 8, **Supplementary Figure S7J**). However, interestingly, downregulation of miR-185-3p expression only partially reversed the effect of circ_0001756 silencing in GC cells. This finding also proves that hsa_circ_0001756 can work in different ways.

### 3.6 Hsa_circ_0001756 binds to PTBP1 to promote its cytoplasmic expression to stabilize PGK1 mRNA

**Figure 4:**
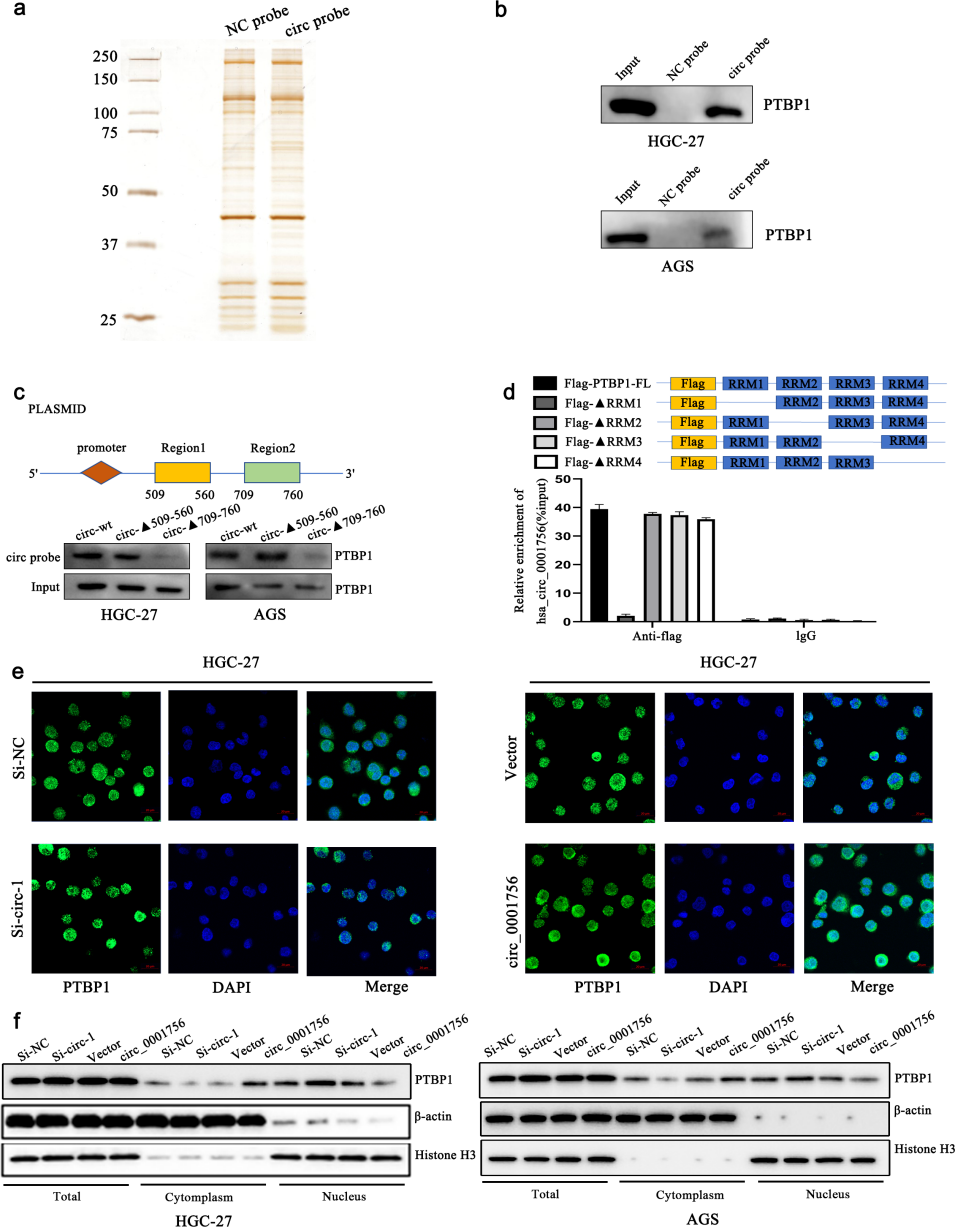
Hsa_circ_0001756 interacts with PTBP1 and promotes its cytoplasmic transport. **(a)** Silver staining of the pulled-down proteins. **(b)** Western blotting confirmed that PTBP1 was enriched in the presence of the hsa_circ_0001756 probe. **(c)** A plasmid expressing a truncated hsa_circ_0001756 fragment was designed (top); the truncated fragment overexpression plasmid was transfected into HGC-27 cells, and an hsa_circ_0001756-specific probe was used for pull-down experiments. **(d)** A Flag-tagged recombinant PTBP1 protein with a truncated RNA recognition sequence was incubated with HGC-27 cell lysates, and the expression of hsa_circ_0001756 was measured by qRT−PCR. **(e)** Immunofluorescence staining of PTBP1 (green) in HGC-27 cells after knockdown or overexpression of hsa_circ_0001756. DAPI (blue) was used for nuclear staining. Original magnification = 400×; scale bar = 20 μm. **(f)** The expression levels of PTBP1 in total lysates and different subcellular fractions were determined by Western blotting. Knockdown or overexpression of hsa_circ_0001756 in HGC-27 and AGS GC cells. The data are shown as the means ± SDs.

RBPs extensively affect gene transcription and translation, and interacting with RBPs is also an important function of circRNAs. Therefore, we further explored whether hsa_circ_0001756 acts through RBPs. To this end, hsa_circ_0001756-bound proteins were detected in the human gastric cancer cell line HGC-27 by an RNA pull-down assay, and the differentially expressed proteins in the pulled down samples were qualitatively analyzed by mass spectrometry (**Figure 4A**) (**Supplementary Table S8**). We also performed GO enrichment analysis of these proteins (**Supplementary Figure S8A**) (**Supplementary Table S9**). We ultimately selected 10 proteins related to translation or mRNA stability (marked in green in **Supplementary Table S8**). According to naive analysis and prediction via RBPsuite (http://www.csbio.sjtu.edu.cn/bioinf/RBPsuite/), there may be 4 proteins that bind to hsa_circ_0001756 (**Supplementary Figure S8B**). Among these, the protein with the highest mass spectrometry score was PTBP1. Therefore, we further investigated whether PTBP1 can bind to hsa_circ_0001756 by RNA pull-down and RIP assays and found that PTBP1 could be pulled down by the hsa_circ_0001756 probe in HGC-27 and AGS cell lysates (**Figure 4B**). Hsa_circ_0001756 could also be precipitated by an anti-PTBP1 antibody (**Supplementary Figure S8C**). We predicted the binding sites between hsa_circ_0001756 and PTBP1 by catRAPID (http://service.tartaglialab.com) and found that the binding sites between hsa_circ_0001756 and PTBP1 are located at 509-560 nt and 709-760 nt (**Supplementary Figure S8D**). We constructed a plasmid expressing hsa_circ_0001756 mutants lacking these two regions and transfected it into cells. A pull-down assay revealed that when the 709-760-nt fragment was removed, the amount of protein pulled down by the PTBP1 probe was significantly reduced, which indicated that PTBP1 mainly bound to hsa_circ_0001756 via this region (**Figure 4C**). To further verify which region of PTBP1 binds to hsa_circ_0001756, we designed PTBP1 mutants in which one of the binding regions was removed. RIP analysis revealed that hsa_circ_0001756 bound mainly to RNA recognition motif 1 (RRM1) of PTBP1 (**Figure 4D**). PTBP1 is a transnucleoprotein with distinct functions in the cytoplasm and nucleus. We found that hsa_circ_0001756 overexpression promoted the export of the PTBP1 protein from the nucleus to the cytoplasm, whereas hsa_circ_0001756 knockdown reduced the cytoplasmic level of PTBP1 (**Figure 4F**). Consistently, immunofluorescence confirmed that hsa_circ_0001756 expression was positively correlated with the level of PTBP1 in the cytoplasm (**Figure 4E**).

**Figure 5:**
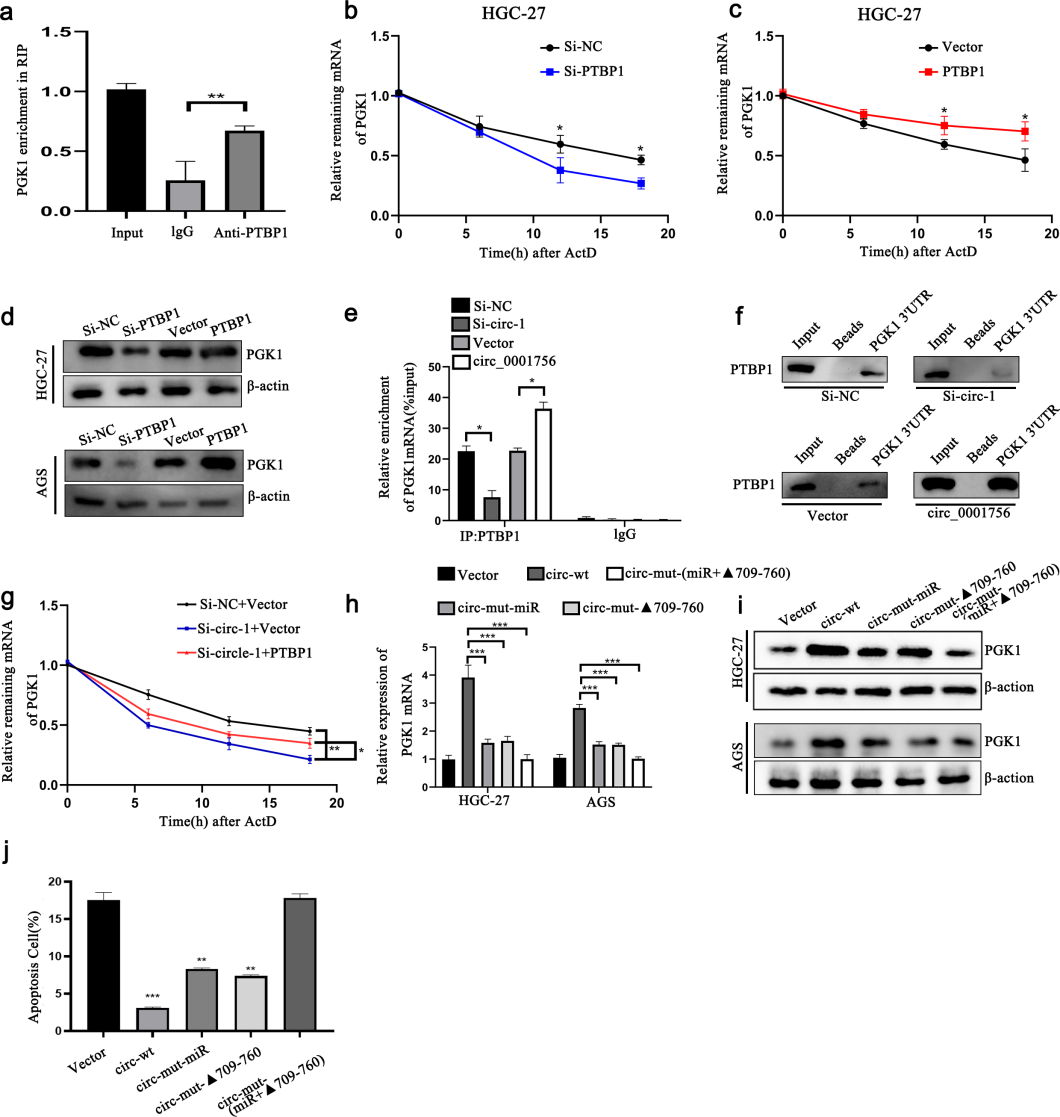
Hsa_circ_0001756 binds to PTBP1 to promote its cytoplasmic expression to stabilize PGK1 mRNA. **(a)** RIP experiments showing that the PTBP1 protein interacts with PGK1 mRNA. **(b, c)** The degradation rate of PGK1 mRNA in HGC-27 cells with PTBP1 overexpression or knockdown at different time points was determined by qRT−PCR. **(d)** Western blot analysis of the protein expression level of PGK1 after dry staining or overexpression of PTBP1 in HGC-27 and AGS GC cells. **(e)** RIP analysis revealed that PGK1 mRNA coprecipitated with PTBP1 after hsa_circ_0001756 was knocked down or overexpressed in HGC-27 cells. **(f)** RNA pull-down analysis of the biotinylated PGK1 3’UTR in HGC-27 cells. Hsa_circ_0001756 siRNA-expressing and hsa_circ_0001756 overexpression plasmids were transfected into HGC-27 cells. **(g)** Degradation rate of PGK1 mRNA in HGC-27 cells transfected with siRNA. **(h, i)** Expression of PGK1 in GC cells transfected with wild-type or mutant hsa_circ_0001756 overexpression plasmids. In circ-mut-miR, the miR-185-3p binding site was mutated, and in circ-mut-Δ709-760; the hsa_circ_0001756 sequence was truncated. circ-mut-(miR + Δ709-760) refers to the mutated miRNA binding site and the truncated portion of the sequence. **(j)** Flow cytometry analysis of apoptosis in HGC-27 cells treated with wild-type or mutant hsa_circ_0001756 overexpression plasmids. The data are shown as the means ± SDs; Student’s t‐test and ANOVA analyzed the difference in **(b, c, e, h, g)**. *p<0.05, **p<0.01, ***p<0.001.

PTBP1 controls the translation and stability of mRNAs. PTBP1 is expressed in almost all human cells and mainly acts as a regulator of glycolysis in tumors. RIP analysis revealed that significantly more PGK1 mRNA was pulled down by an anti-PTBP1 antibody than by IgG (**Figure 5A**). In the experiment involving ActD, knockdown of PTBP1 reduced the mRNA level of PGK1 and shortened the half-life of the PGK1 transcript (**Figures 5B–D**). We then verified whether hsa_circ_0001756 affects the binding of PTBP1 to PGK1. A RIP assay revealed that inHGC-27 cells, knockdown of hsa_circ_0001756 significantly reduced the binding of PTBP1 to PGK1 (**Figure 5E**). Pull-down experiments also verified that reducing the expression of hsa_circ_0001756 reduced the binding of PTBP1 to the 3’UTR of PGK1 (**Figure 5F**). Rescue experiments revealed that the reduction in PGK1 mRNA stability caused by hsa_circ_0001756 knockdown in HGC-27 cells was reversed after PTBP1 overexpression (**Figure 5G**). We then further verified whether hsa_circ_0001756 can act through ceRNAs and RBPs simultaneously. Mutant miR-185-3P (circ-mut-miR) and miR-185-3P lacking the PTBP1-binding region (circ-mut-Δ709-760) were transfected into HGC-27 cells. The results showed that circ-mut-miR and circ-mut-Δ709-760 could still partially increase the expression of PGK1. However, circ-mut-(miR+▴709-760) did not increase the level of PGK1 (**Figures 5H, I**). Flow cytometry analysis of apoptosis also verified that circ-mut-miR and circ-mut-▴709-760 partially inhibited apoptosis, whereas circ-mut-(miR+▴709-760), similar to the empty vector, did not inhibit apoptosis (**Figure 5J**)(**Supplementary Figure S8E**). Therefore, we found that hsa_circ_0001756 not only acts through ceRNAs but also regulates the level of PGK1 through PTBP1.

### 3.7 Hsa_circ_0001756 promotes tumorigenesis *in vivo*

**Figure 6:**
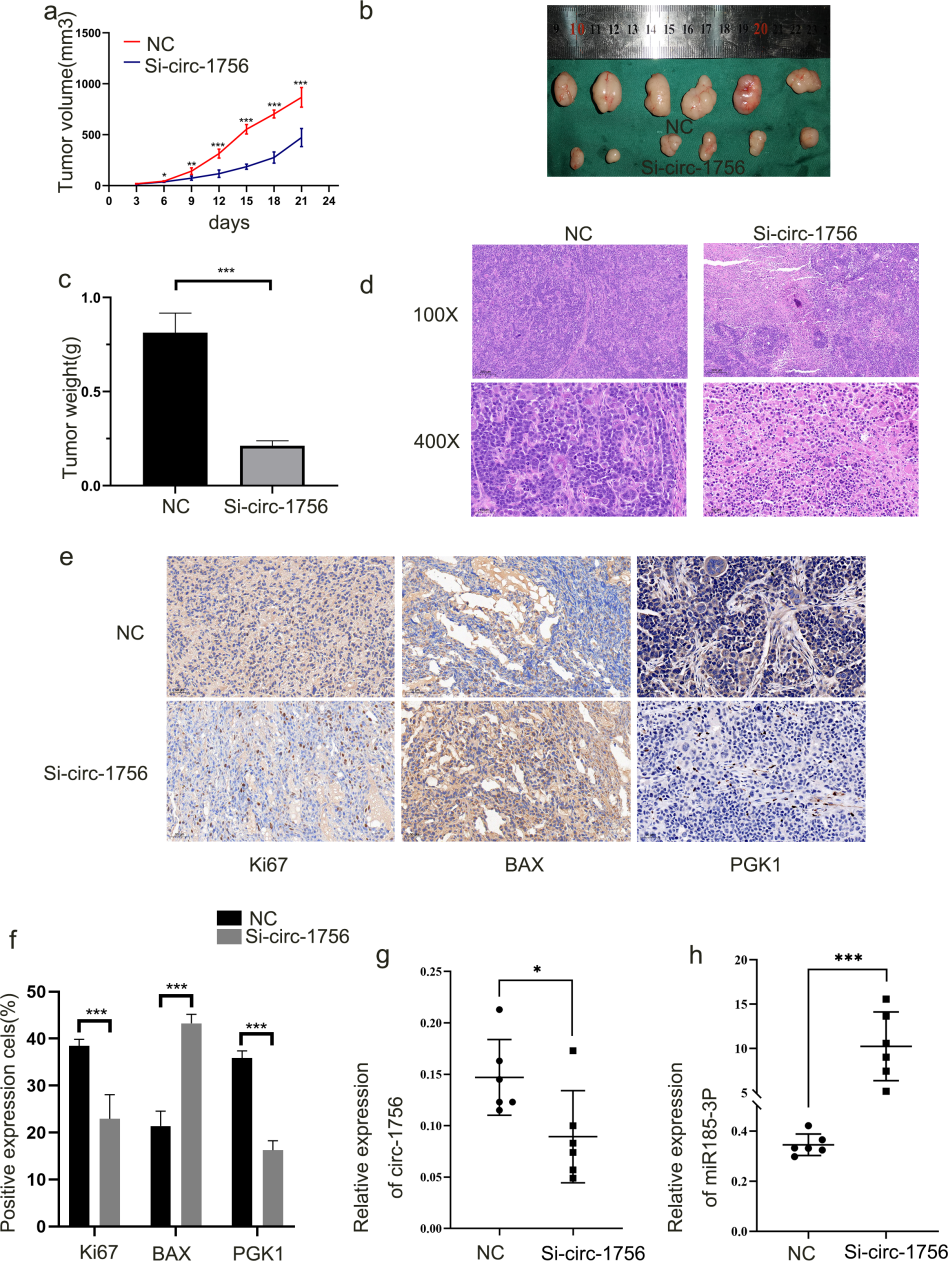
Hsa_circ_0001756 promotes tumorigenesis *in vivo* **(a)** Nude mice were subcutaneously injected with HGC-27 cells stably transfected with hsa-circ_0001756 siRNA (Si-circ_0001756) (right) or control siRNA (Si-NC) (left). After 3 days, the sizes of the subcutaneous tumors were measured every 3 days, and the tumor volume was calculated. **(b)** The tumors were dissected. A ruler was used to determine the size of the tumor. **(c)** Tumor weights in the different treatment groups. **(d)** Hematoxylin and eosin (HE) staining of tumors was performed. **(e)** IHC analysis of the expression of PGK1;Bax and Ki67 in tumors (400x magnification, scale bars = 50 μm). **(f)** Statistical analysis of the percentage of IHC-positive cells in the tumors. **(g)** qRT−PCR analysis of hsa_circ_0001756 expression in tumor tissues subjected to different treatments. **(h)** qRT–PCR analysis of miR-185-3p expression in tumor tissues subjected to different treatments. The data are shown as the means ± SDs; Student’s t‐test and ANOVA analyzed the difference in **(a–c, f–h)**. *p<0.05, **p<0.01, ***p<0.001.

To determine whether hsa_circ_0001756 promotes the development of GC *in vivo*, HGC-27 cells with stable hsa_circ_0001756 knockdown were injected subcutaneously into nude mice. The mean tumor size and mean tumor weight (**Figure 6C**) in the hsa_circ_0001756 silencing group were significantly reduced (**Figures 6A, B**). HE staining revealed the presence of solid tumors (**Figure 6D**). The hsa_circ_0001756 silencing group presented a significantly reduced level of hsa_circ_0001756 in tumors according to qRT−PCR (**Figure 6G**), whereas miR-185-3p expression increased (**Figure 6H**). This finding is consistent with the results from cells and tissues. Immunohistochemical analysis of tumors from mice revealed that hsa_circ_0001756 silencing reduced the level of PGK1 and Ki67, Increased level of Bax(**Figures 6E, F**). In conclusion, interfering hsa_circ_0001756 level effectively inhibited GC cell growth *in vivo*.

## 4 Discussion

Research on the effects of ceRNAs on tumor glycolysis has gradually increased in recent years. Qiong Chen et al. demonstrated that hsa_circ_0001756 competitively inhibits miR-27a-3p and thereby affects tumor function (29). However, how circRNAs act as ceRNAs to regulate glucose and energy metabolism in tumors is unclear. Noncoding RNAs can regulate target genes and function in tumors through various mechanisms. For example, they can act as sponges for miRNAs or proteins or have a coding function. We investigated the different modes of action of hsa_circ_0001756 in GC. CeRNAs bind and inhibit miRNA expression via miRNA response elements (MREs), thereby affecting the expression of target genes closely related to tumor regulation (30). CircRNAs (22) and lncRNAs (31) can act as ceRNAs (32). CircRNAs are noncoding RNA molecules that do not have a 5’ end cap or a 3’ end poly(A) tail and form a covalent loop (33). CircRNAs are highly conserved in different tissues, their ring structure is stable, and they are highly resistant to nucleases, so their levels can sometimes be more than 10 times higher than those of linear RNAs; MREs are abundant throughout circRNAs, and one circRNA can bind multiple miRNAs at the same time (34). These unique characteristics allow circRNAs to function as ceRNAs. Our research focused on hsa_circ_0001756. Hsa_circ_0001756 consists of a loop of exon 2 of the HIPK2 gene, located on chromosome 7, and is 1084 bases long. hsa_circ_0001756 can regulate various functions, such as tumor proliferation (35), but whether hsa_circ_0001756 exerts this effect in GC is unclear. In our study of 74 pairs of GC tissues, we found that the level of hsa_circ_0001756 in cancer tissues was greater than that in control tissues (P=0.0013). Hsa_circ_0001756 is more stable than linear RNA is and cannot be easily degraded by RNase R. In addition, hsa_circ_0001756 promoted glycolysis and proliferation in GC cells, as shown by a series of *in vitro* functional assays. Hsa_circ_0001756 was also proven to promote cancer in nude mice *in vivo*. In addition, Yang Cao et al. (36) reported that hsa_circ_0001756 promotes cisplatin (DDP) resistance and malignant behavior in DDP-resistant ovarian cancer cells, revealing that hsa_circ_0001756 has cancer-promoting effects in different types of tumors.

We used FISH to verify that hsa_circ_0001756 is present in the cytoplasm and that circRNAs usually act as miRNA sponges to inhibit the function of miRNAs and thus protect target proteins (32). For example, circRNA_0005529 promotes the growth and metastasis of GC by regulating the miR-527/Sp1 axis (37), and circRNA hsa-circ_0001017 inhibits GC progression by acting as a sponge for miR-197 (38). CircRNA_0005529 promotes epithelial−mesenchymal transformation in GC by regulating miR-527/Sp1 (37). We successfully amplified hsa_circ_0001756 after immunoprecipitation of the agO2-mirNA-circrNA complex. In addition, we verified the colocalization of hsa_circ_0001756 and miR185-3p in the cytoplasm of GC cells by FISH and confirmed the mutual binding of hsa_circ_0001756 and miR-185-3p through the use of a dual-luciferase reporter. Previous studies revealed that hsa_circ_0001756 expression is negatively correlated with miR-185-3p expression. In the ceRNA regulatory network, miRNAs play key roles as bridges. MiRNAs can regulate cellular functions by binding to the 3’-UTRs of target genes (39).

The abnormal expression of miR-185-3p is related to cancer progression (40, 41), but its function in GC is not yet known. We explored how miR-185-3p is involved in GC. Our research revealed that increasing the expression of miR-185-3p inhibited proliferation, migration, invasion and glucose metabolism in GC cells, indicating that miR-185-3p can inhibit GC progression. We also found that miR-185-3p functions mainly by targeting PGK1. In recent years, the regulation of PGK1 by noncoding RNAs has received increasing attention. PGK1 is the only enzyme encoded by an X-linked gen, and participates in the first step of ATP production via the glycolytic pathway (42). In GC cells, PGK1 protein expression was increased by overexpression of hsa_circ_0001756 and repressed by miR-185-3p. Therefore, circ_0001756 can adsorb miR-185-3p to induce the expression of PGK1. We can design new therapeutics targeting this effect to treat GC. We subsequently conducted a series of rescue experiments to explore how hsa_circ_0001756 regulates PGK1 through miR-185-3p to affect the biological functions of GC. By analyzing Pita, RNAhybrid, and miRanda databases, predict miRNA binding sites containing miR-185-3p in hsa_circ_0001756. FISH, Experiments such as biotin labeled probe pull-down analysis, dual luciferase reporter gene analysis, and RIP analysis have confirmed that hsa_circ_0001756 and miR-185-3p are co localized in the cytoplasm, hsa_circ_0001756s directly binds to miR-185-3p. Subsequent reaction experiments further confirmed that hsa_circ_0001756 reversed the carcinogenic effect of miR-185-3p. The upregulation of miR-185-3p inhibits the level of PGK1, indicating that miR-185-3p is a key negative regulator of PGK1. Our research results provide sufficient evidence that hsa_cir-0001756, as a miR-185-3p sponge, greatly enriches the regulatory mechanism of cancer glycolysis and provides a theoretical basis for designing molecular targeted drugs targeting tumor energy metabolism pathways in the future.

RBPs are essential regulators of transcription and translation. CircRNAs bind to these genes and then regulate downstream genes. Multiple circRNAs as ceRNAs and RBPs. RBPs can regulate gene expression posttranscriptionally (43). We found that hsa_circ_0001756 could stabilize PGK1 mRNA by binding PTBP1 to promote its cytoplasmic expression. PTBP1 is located on human chromosome 19p13.3 and has a nuclear shuttle domain and 4 RRM domains (44). This protein shuttles between the cytoplasm and nucleus and performs different functions (45). The upregulation of PTBP1 expression is related to the proliferation and migration of various tumors (1, 46), and glycolysis is the most important mechanism in tumor progression (47). We found that when PTBP1 is localized in the cytoplasm, it can significantly increase the stability of PGK1 mRNA and that PTBP1 mainly interacts with PGK1 through the RRM1 domain. Therefore, we found that the RRM1 domain of PTBP1 plays an important role in the function of hsa_circ_0001756 in GC progression.

Glycolysis is the main pathway for providing energy to tumor cells. There is a synergistic effect of multiple enzymes in the process of glycolysis, and some circRNAs can affect these enzymes to regulate glycolysis. These circRNAs may induce Warburg effect in tumor tissues. Recently, it has been found that transcription factors (TFs) also have an impact on the Warburg effect (48). CircRNAs can regulate the ability of TFs to express abnormally in tumors. CircRNA can affect target TFs such as HIF-1, CUX1, C-myc, etc. by targeting miRNAs. These circRNAs TFs, The signal pathway network affects the Warburg effect. As one of the TFs and important oncogenes, myc and hif-1 can also activate the Warburg effect (49). The abnormally expressed HIF-1 and c-myc affect tumor glycolysis by acting on enzymes in the glycolysis pathway. Glycolysis plays an important role in tumor cells, and the specific glycolysis mode of tumor cells can inhibit immune cell function, leading to immune escape; Metabolites related to glycolysis can regulate immune responses; Meanwhile, the self glycolysis of immune cells affects anti-tumor immunity. Inhibiting tumor cell glycolysis can enhance immune cell function; Increase the secretion of co stimulatory factors. Our research enriches the key circRNAs in the glycolysis pathway of gastric cancer, providing new ideas for future immunotherapy.

## 5 Conclusions

**Figure 7:**
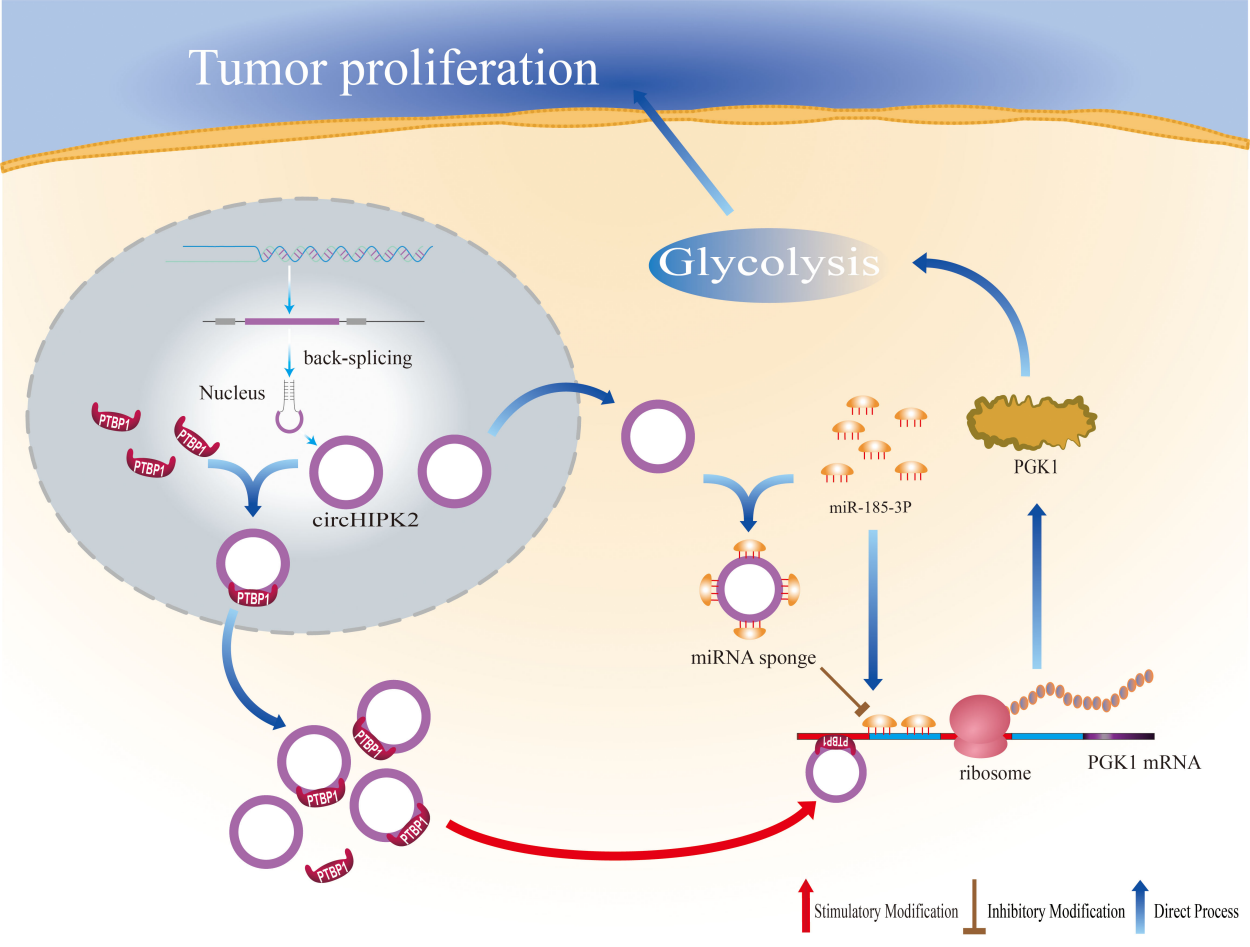
Schematic diagram illustrating the mechanism underlying the oncogenic function of hsa_circ_0001756.

In short, hsa_circ_0001756 is overexpressed in tumors and often indicates a poor prognosis in GC patients. Our study revealed that hsa_circ_0001756 plays a regulatory role in two different ways: hsa_circ_0001756 drives GC progression by increasing PGK1 mRNA expression and stability in a ceRNA- and RBP-dependent manner (**Figure 7**). Therefore, the regulatory role of hsa_circ_0001756 in glycolysis is a novel therapeutic target for GC.

## Data Availability

The datasets presented in this study can be found in online repositories. The names of the repository/repositories and accession number(s) can be found in the article/**Supplementary Material**.
