# Supplementary material for: Hsa_circ_0001756 drives gastric cancer glycolysis by increasing the expression and stability of PGK1 mRNA
Source: Front Immunol. 2025 Feb 20;16:1511247. doi: 10.3389/fimmu.2025.1511247 (PMC11882586; doi:10.3389/fimmu.2025.1511247)
Supplement: Supplementary file 1 [file DataSheet1.docx]

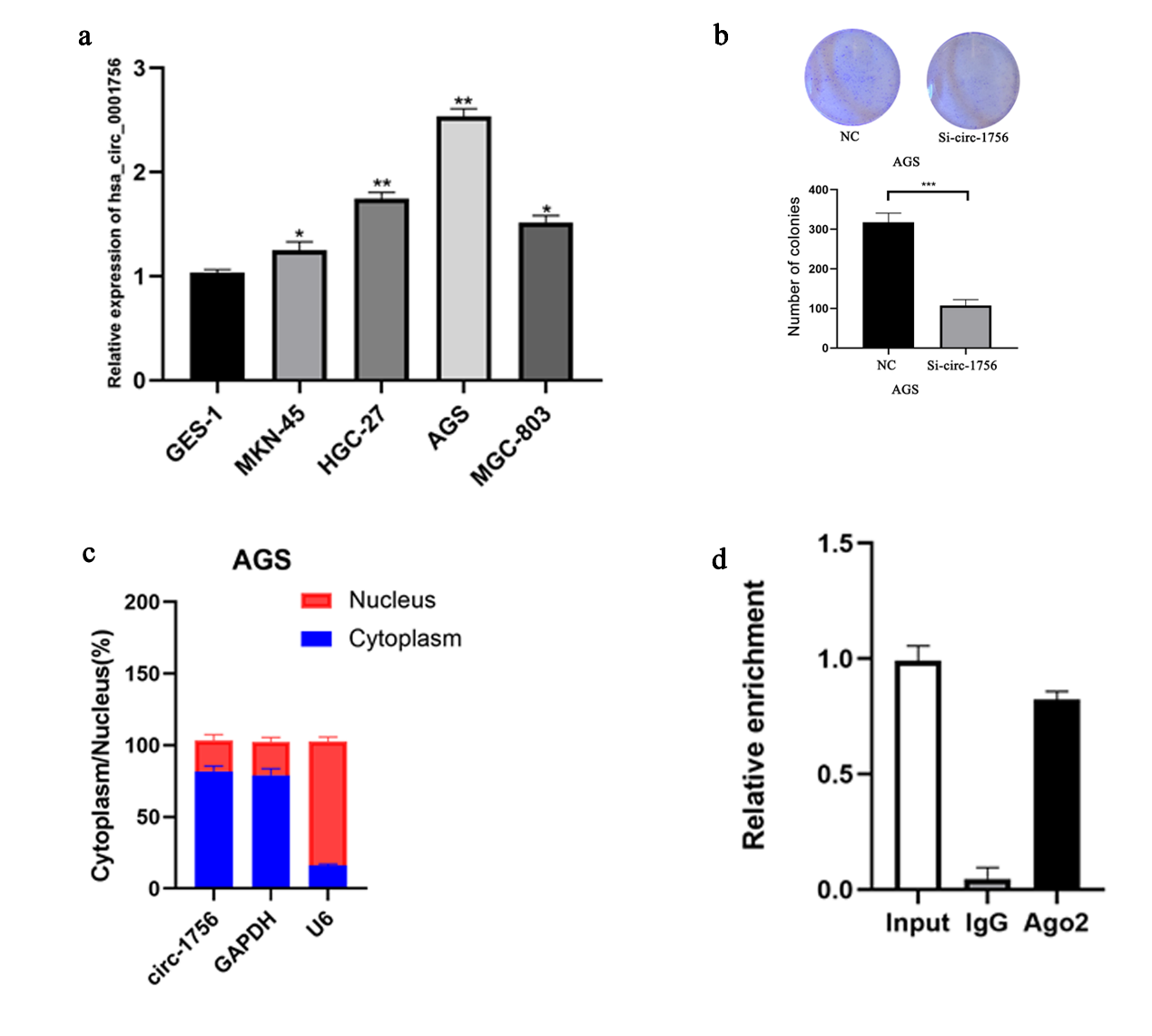


Additional file 2: Figure S1a. qRT-PCR analysis of the expression of hsa_circ_0004872 in different GC cells.; b. The cytoplasmic and nuclear mRNA fractionation experiments showed the location of hsa_circ_0001756 in AGS cell lines. U6 and GAPDH were used as positive controls in the nucleus and cytoplasm, respectively.c. U6 and GAPDH were used as positive controls in the nucleus and cytoplasm, respectively .d. RIP analysis of hsa_circ_0001756 levels in immunoprecipitates obtained with an anti-AGO2 antibody from GC cells. Data are shown as mean ± SD;Student's t‐test and ANOVA analysed the difference in a-d. *p<0.05, **p<0.01, ***p<0.001.


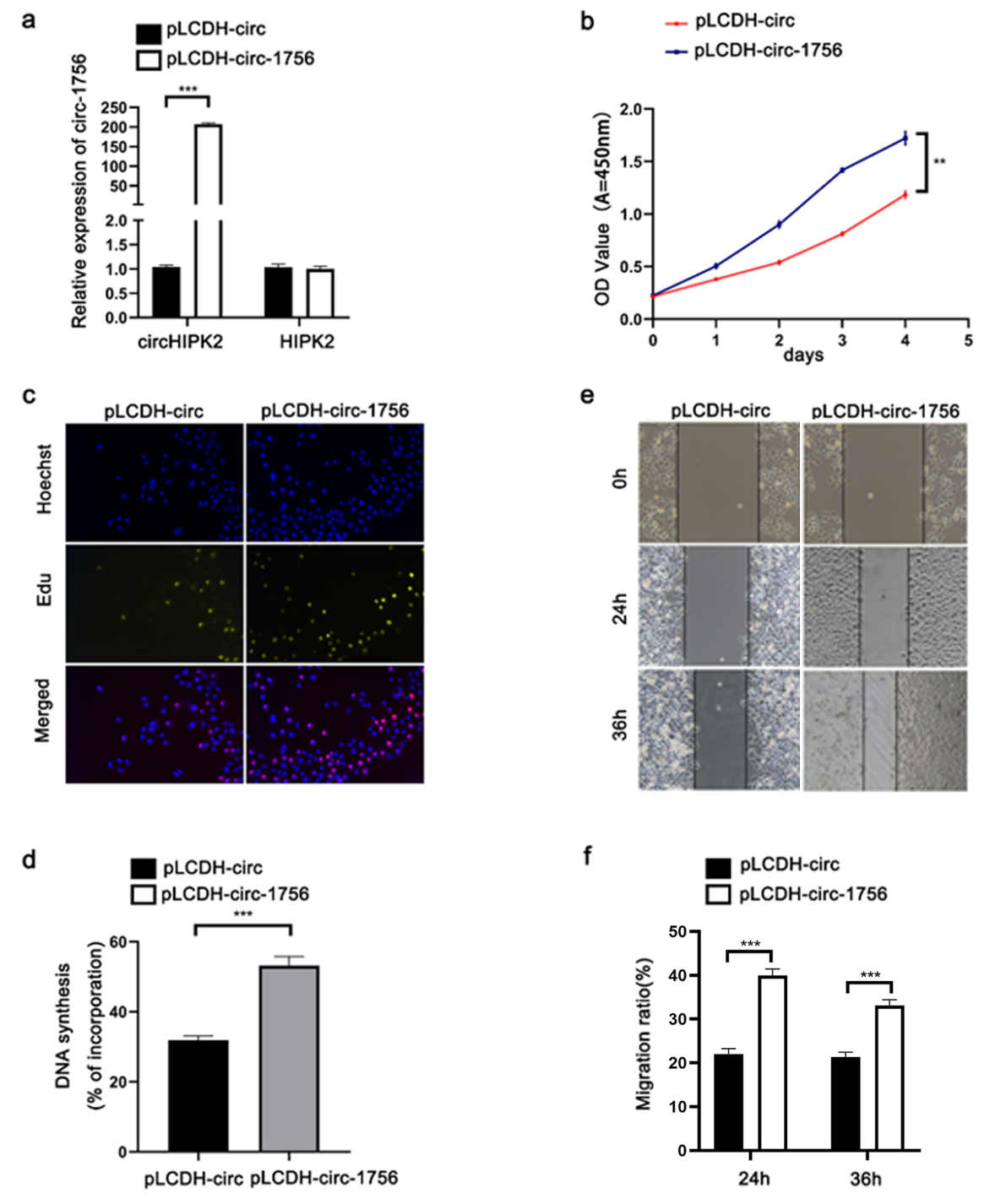


Additional file.3:Figure S2.hsa-circ_0001756 overexpression promotes the proliferation and migration of GC: qRT–PCR analysis of hsa-circ_0001756 (circHIPK2) and linear HIPK2 (HIPK2) in AGS cells transfected with hsa-circ_0001756 overexpression vector (pLCDH-circ-1756) and control vector (pLCDH-NC). b: CCK-8 analysis of the cell proliferation ability of different groups of transfected AGS cells. c: EdU analysis of the proliferation ability of different groups of transfected AGS cells. Scale bar: 50 μm. d: Statistical analysis of the proportion of EdU-positive cells in different groups of transfected AGS GC cells. e: Scratch healing test of different groups of transfected AGS cells. Scale bar: 50 μm. f: Statistical analysis of cell migration in the scratch wound healing test in different groups of transfected AGS GC cells. Data are shown as mean ± SD; Student's t‐test and ANOVA analysed the difference in a-b, e-f. *p<0.05, **p<0.01, ***p<0.001.


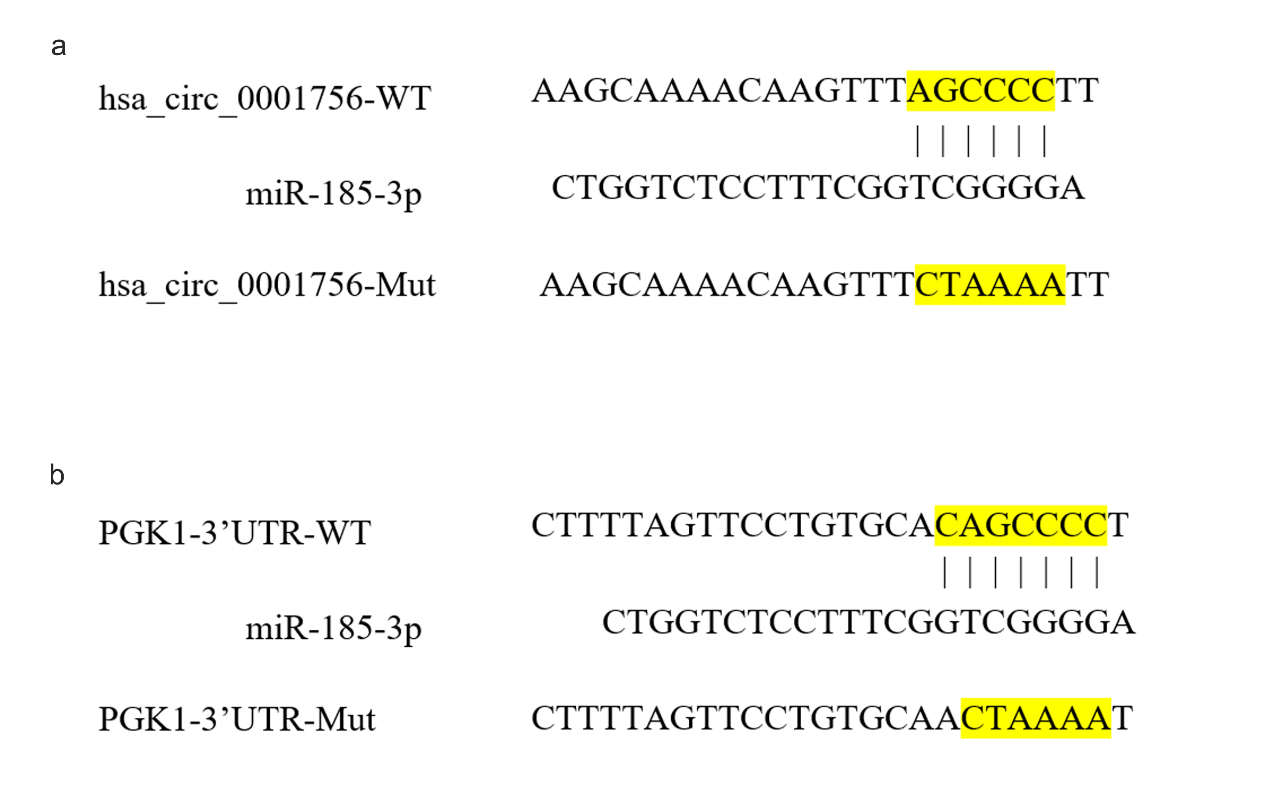


Additional file 4:Figure S3. Schematic diagam of dual luciferase vector. (a) Schematic diagam of dual luciferase vector pMIRGLO-circ_1756-WT/Mut. the predicted complementary sequences of miR-185-3p in the sequences of hsa_circ_0001756.(b)(B) Schematic diagam of dual luciferase vector pMIR-PGK1-WT/Mut. the predicted complementary sequences of miR-185-3p in the 3’UTR of PGK1.


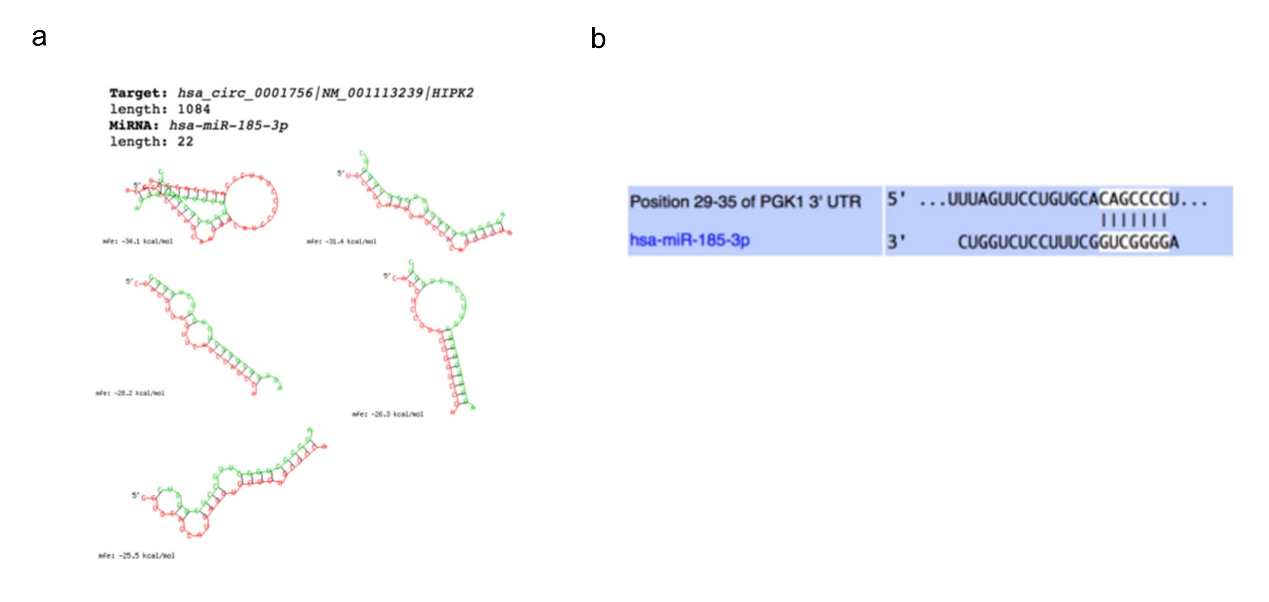


Additional file 5:Figure S4. Predictive analysis of circHIPK2 / Mir-185-3p/PGK1 regulatory axis.a: RNAhybrid predictive analysis hsa_circ_0001756 and miR-185-3p have 8 stable binding (maximum binding free energy (MFE) &gt; -20kcal/mol), and the figure shows the second conformation of its binding region (the first 5 are listed).b: Targetscan information analysis software predicted the binding sequence of Mir-185-3p and PGK1 3 'UTR.


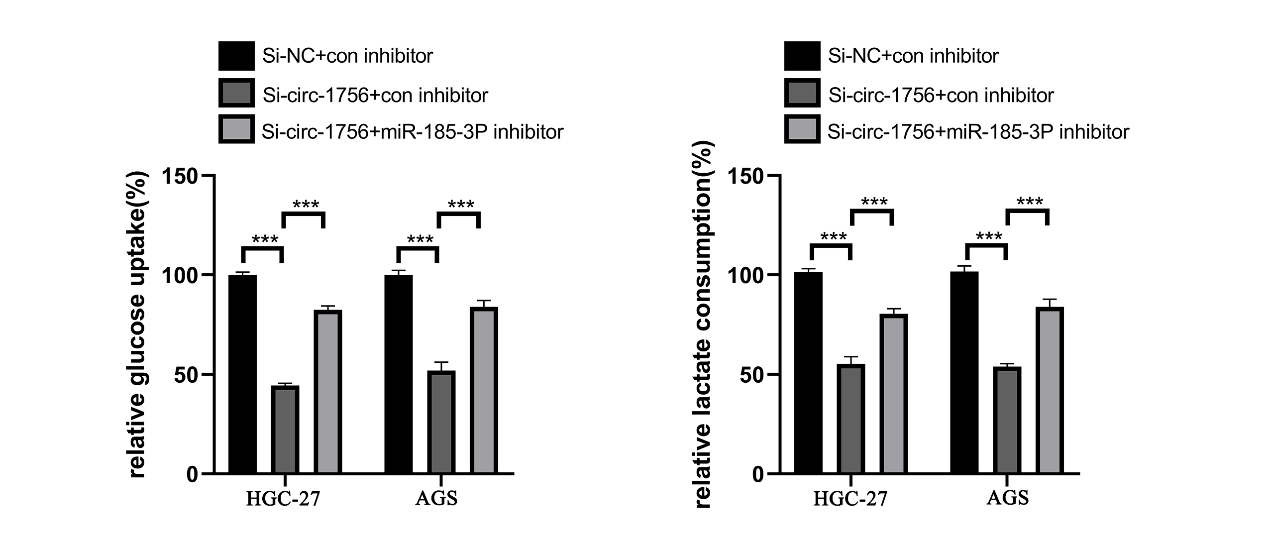


Additional file 6:Figure S5. Downregulation of miR-185-3p reversed the effect of circ_0001756 silencing on glycolysis in GC cells.a: The relative glucose uptake of AGS and HGC-27 cells was measured after transfection.b: The relative lactate production of AGS and HGC-27 cells was detected after transfection. Data are shown as mean ± SD; Student's t‐test and ANOVA analysed the difference .*p<0.05, **p<0.01, ***p<0.001.


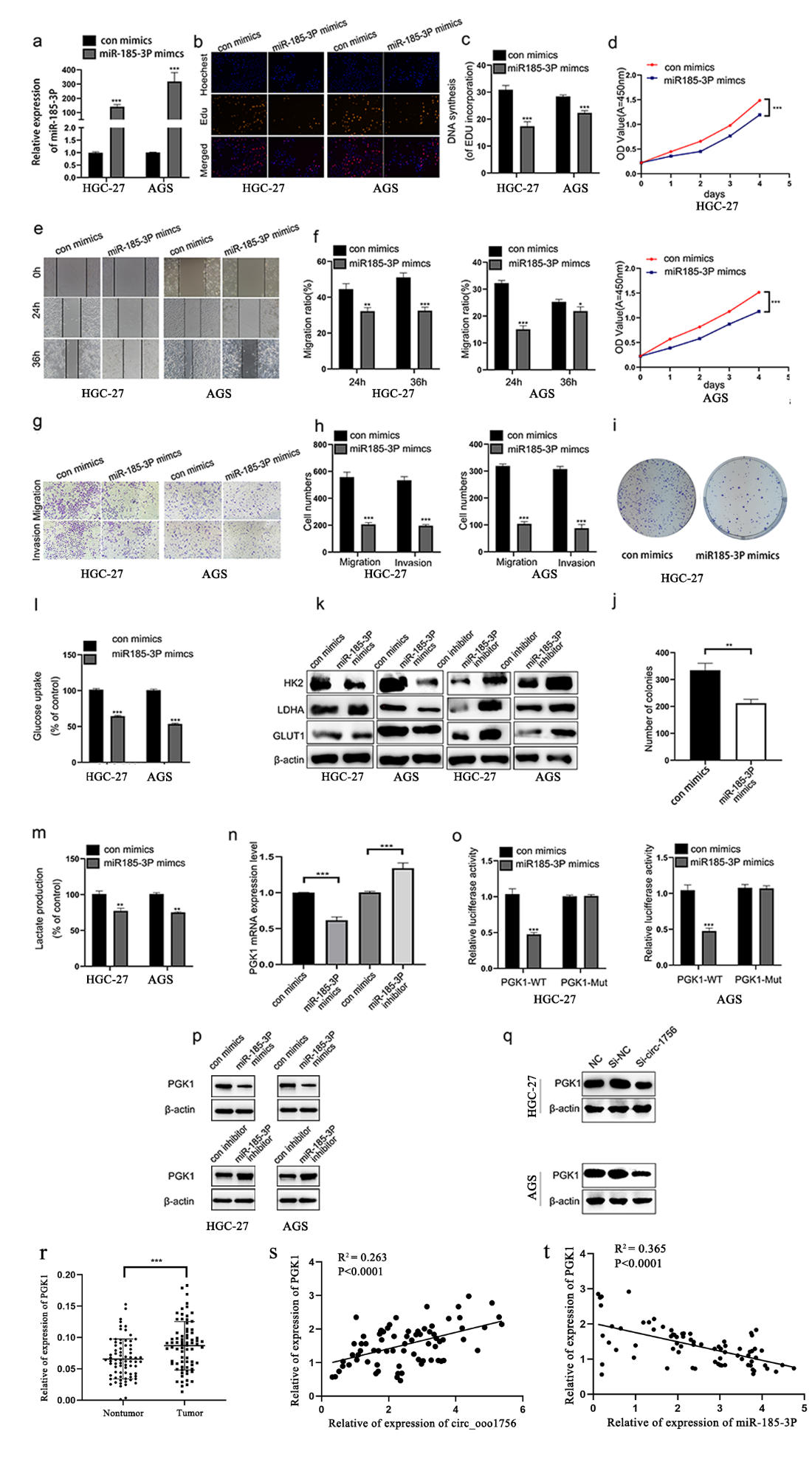


Additional file 7 Figure S6. Inhibition of miR-185-3p increases glycolysis through PGK1.(a): qRT–PCR analysis of miR-185-3p levels in AGS and HGC-27 cells transfected with miR-185-3p mimic or control mimic.(b): EdU analysis of the proliferation of AGS and HGC-27 cells transfected with miR-185-3p mimic or control mimic. Scale bar: 50 μm.(c): Statistical analysis of the proportion of EdU-positive cells in transfected GC cells.(d): CCK-8 analysis of the proliferation ability of AGS and HGC-27 cells transfected with miR-185-3p mimic or control mimic.(e): Cell proliferation and scratch healing analysis of AGS and HGC-27 cells transfected with miR-185-3p mimic or control mimic. Scale bar: 50 μm.(f): Statistical analysis of cell migration in the scratch wound healing test.(g): Transwell invasion and migration assay of AGS and HGC-27 cells transfected with miR-185-3p mimic or control mimic. Scale bar: 50 μm.(h): Statistical analysis of the number of cells passing through the Transwell chamber in the transfected AGS and HGC-27 cells. All data are mean ± SD.(i): Analysis of the proliferation and colony formation of AGS and HGC-27 cells transfected with miR-185-3p mimic or control mimic.(j): Statistical analysis of the colony formation rate in transfected GC cells.(k): The protein abundance of HK2, LDHA and GLUT1 in AGS and HGC-27 cells transfected with miR-185-3p mimic or control mimic was analysed by Western blotting.(l): Relative glucose uptake was detected in AGS and HGC-27 cells transfected with miR-185-3p mimic or control mimic.(m): Relative lactate production was detected in AGS and HGC-27 cells transfected with miR-185-3p mimic or control mimic.(n): qRT–PCR analysis of PGK1 mRNA expression levels in transfected GC cells.(o): AGS and HGC-27 cells were cotransfected with PGK1-WT or PGK1-Mut reporter gene constructs with control or miR-185-3p mimics, and dual-luciferase reporter activity was measured 48 hours after transfection.(p): Western blot analysis of PGK1 protein expression levels in AGS and HGC-27 cells transfected with miR-185-3p mimic or control mimic.(q): Western blot analysis of PGK1 protein levels in AGS cells transfected with hsa_circ_0001756 siRNA. （r）: RT-PCR was used to detect the expression of PGK1 in 74 pairs of GC patients and their adjacent normal tissues. (s): PGK1 is positively correlated with circHIPK2. (t): Correlation analysis shows that miR185-3p is negatively correlated with the expression level of PGK1. Data are shown as mean ± SD; Student's t‐test and ANOVA analysed the difference in a, c, d, f, h, j , l-o.*p<0.05, **p<0.01, ***p<0.001.


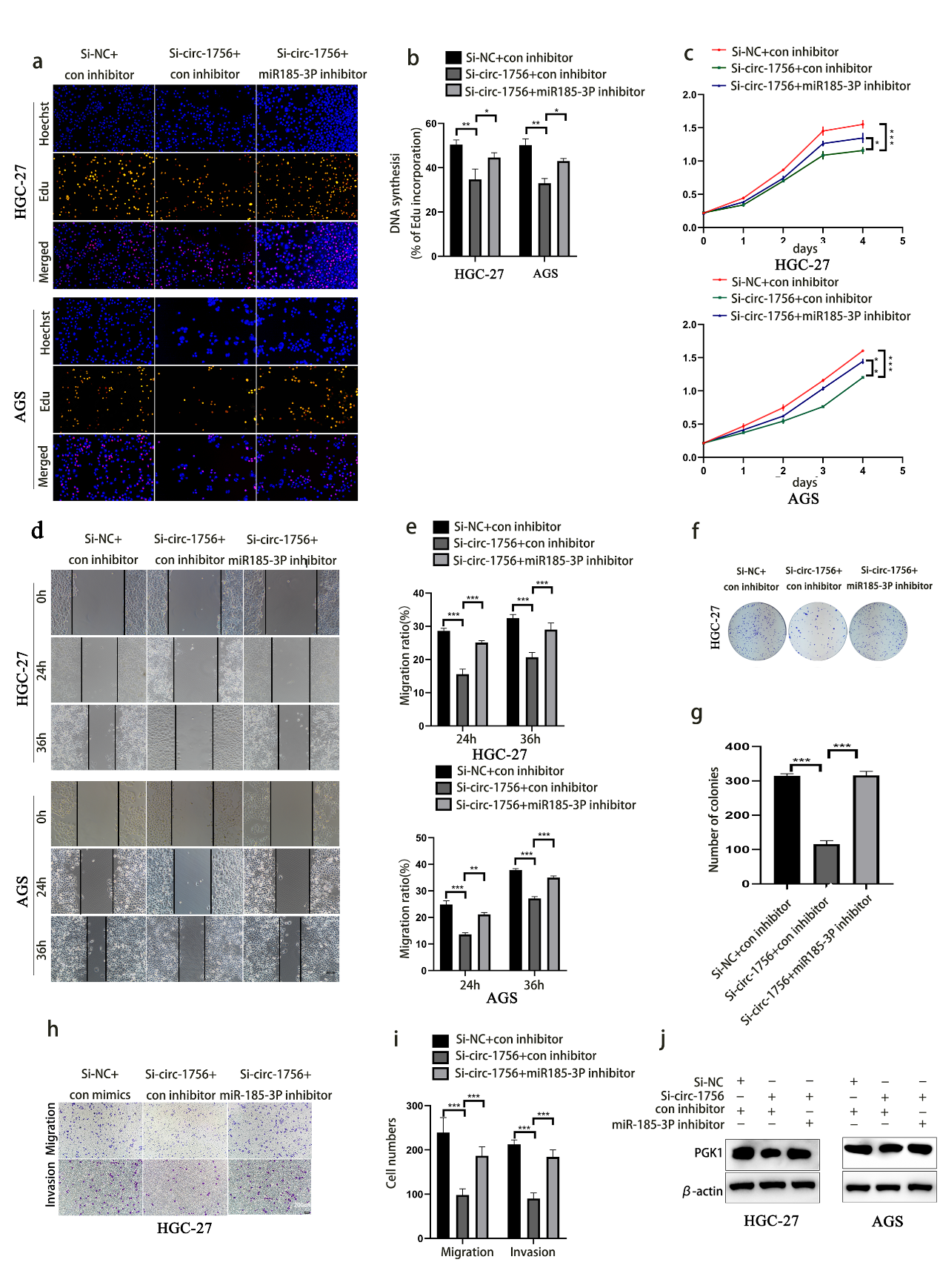


Additional file 8 Figure S7. Downregulation of miR-185-3p expression reverses the effect of circ_0001756 silencing in GC cells. (a): Transfection of Si-NC + control mimic or hsa-circ_0001756 siRNA (Si-circ-1756) + control mimic or Si-circ-1756 + miR was performed; EdU analysis of the proliferation ability of HGC-27 and AGS cells cotransfected with miR-185-3p inhibitor. Scale bar: 50 μm.(b): Statistical analysis of the proportion of EdU-positive cells among different groups of cotransfected GC cells.(c): CCK-8 analysis of the cell proliferation ability of cotransfected AGS and HGC-27 cells.(d): Scratch healing test of different cotransfected AGS and HGC-27 cells. Scale bar: 50 μm.(e): Statistical analysis of cell migration in the scratch wound healing test.(f): Analysis of the proliferation/colony formation of different groups of cotransfected HGC-27 cells.(g): Statistical analysis of the colony formation rate in transfected GC cells.(h): Transwell invasion and migration assays of treated HGC-27 cells. Scale bar: 50 μm.(i) Statistical analysis of the number of cells passing through the Transwell chamber in the processed HGC-27 cells.(j): Western blot analysis of PGK1 protein expression levels in different groups of cotransfected AGS and HGC-27 cells.Data are shown as mean ± SD; Student's t‐test and ANOVA analysed the difference in b-c, e, g,i. *p<0.05, **p<0.01, ***p<0.001.


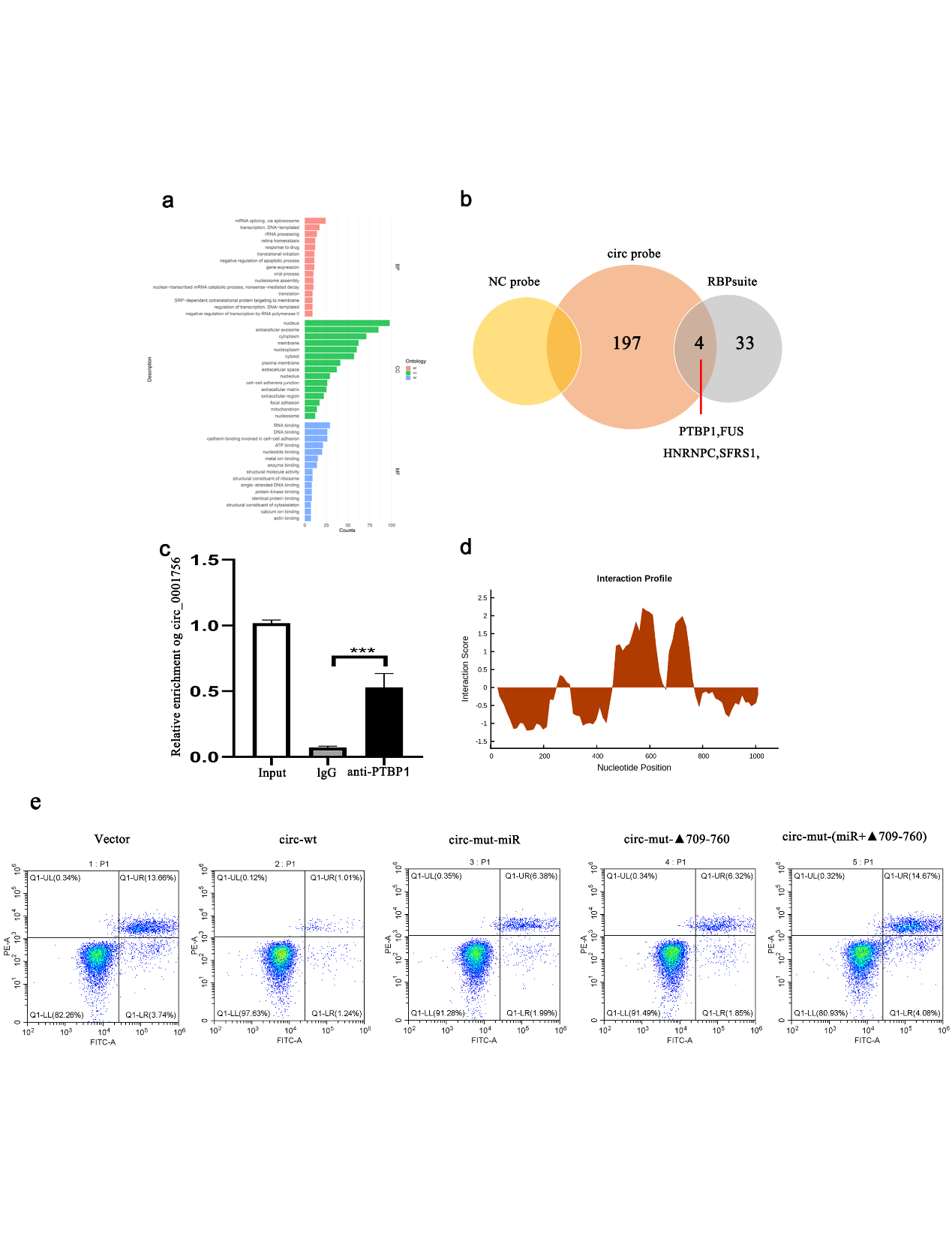


Additional file 9 Figure S8. (a): GO analysis. (b): Venn diagram analysis of RBPsuite and pull-down predicted RBPs of hsa_circ_0001756.(c): RIP analysis showed that PTBP1 protein could co-precipitate with hsa_circ_0001756. (d): catRAPID website predicts hsa_circ_0001756 and PTBP1 binding sites. (e): Flow cytometry of apoptosis in HGC-27 cells treated with wild-type or mutant hsa_circ_0001756 overexpression plasmids.Data are shown as mean ± SD; Student's t‐test and ANOVA analysed the difference in c.*p<0.05, **p<0.01, ***p<0.001.
