# Supplementary figures and images for: Hsa_circ_0001756 drives gastric cancer glycolysis by increasing the expression and stability of PGK1 mRNA
Source: Front Immunol. 2025 Feb 20;16:1511247. doi: 10.3389/fimmu.2025.1511247 (PMC11882586; doi:10.3389/fimmu.2025.1511247)

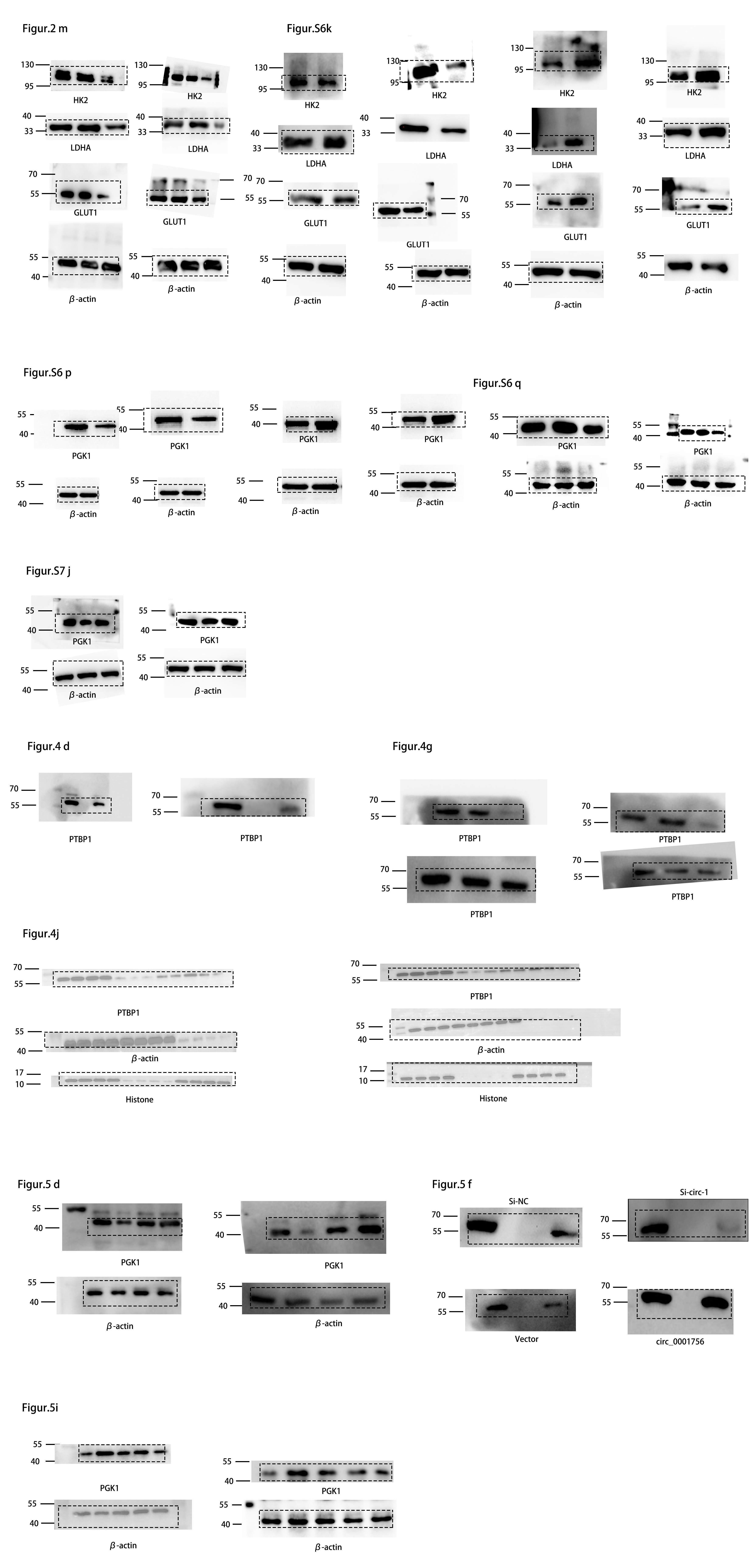

Supplement: Supplementary file 2 [file Image1.tif]
