# Supplementary material for: Hsa_circ_0001756 drives gastric cancer glycolysis by increasing the expression and stability of PGK1 mRNA
Source: Front Immunol. 2025 Feb 20;16:1511247. doi: 10.3389/fimmu.2025.1511247 (PMC11882586; doi:10.3389/fimmu.2025.1511247)
Supplement: Supplementary file 3 [file Table1.docx]

**Additional file 1 legends,**

**Table S1,** Primers and RNA sequences used in this study.

**Table S2,** SiRNA sequences used in this study.

**Table S3,** MiRNA mimics, and inhibitors sequences used in this study.

**Table S4,** Biotinylated probes sequences used in this study.

**Table S5,** FISH probes sequences used in this study.

**Table S6,** Pull down probes sequences used in this study.

**Table S7,** Patients and tumor characteristics, hsa_circ_0001756 expression in GC samples

**Table S8,** Pull-down differential proteins

**Table S9,** GO analysis

**Table S10,**Predicted miRNAs with potential binding ability with hsa_circ_0004872 in different databases.

**Table S1**

| **Primer sequence** |  |  |
| --- | --- | --- |
| CircHIPK2 | Forward | CAACCATACAGAGTCAAGGTCATCG |
| CircHIPK2 | Reverse | TCACACTACAGAAGGCACTTGATTG |
| HIPK2 | Forward | AGCCTACCTTACGAGCAGACCATC |
| HIPK2 | Reverse | CAGTGCTTCGACGCATTAGGTTG |
| GAPDH | Forward | GAACGGGAAGCTCACTGG |
| GAPDH | Reverse | GCCTGCTTCACCACCTTCT |
| Divergent GAPDH | Forward | GAAGGTGAAGGTCGAGTC |
| Divergent GAPDH | Reverse | GAAGATGGTGATGGGATTTC |
| U6 | Forward | CTCGCTTCGGCAGCACA |
| U6 | Reverse | AACGCTTCACGAATTTGCGT |
| β-actin | Forward | CTCCATCCTGGCCTCGCTGT |
| β-actin | Reverse | GCTGTCACCTTCACCGTTCC |
| PGK1 | Forward | AAGAAGTTGAACGAGTGGTTGG |
| PGK1 | Reverse | GCCCTGTTTACTGCTCTCCC |
| PTBP1 | Forward | TTTTCCAAGCTCACCAGCCT |
| PTBP1 | Reverse | TATACCAGGTGCACCGAAGG |
| miR-139-3P | Forward | AAGCCCTTACCCCAAAAAGTAT |
| miR-139-3P | Reverse | CTTTTTGCGGTCTGGGCTTGC |
| miR-448 | Forward | CGGGGCAGCUCAGUACAG |
| miR-448 | Reverse | CAGTGCGTGTCGTGGAGT |
| miR-370-3P | Forward | GCCTGCTGGGGTGGAACCTGGT |
| miR-370-3P | Reverse | General downstream primer' |
| miR-3131 | Forward | AAGGCCCTTCCACCAGTCCTCGA |
| miR-3131 | Reverse | CCGAGGACTGATGGAAGATTCC |
| miR-185-3P | Forward | ACACTCCAGCTGGGTGGAGAGAAAGGCAGT |
| miR-185-3P | Reverse | ACTGACTGATGCAATCTCAACTGGTGTCGTGGA |
| Has-circ-0001756 (Divergent primer) | Forward | ACTGCCACCTCCAAAAACAG |
| Has-circ-0001756 (Divergent primer) | Reverse | TCGTAGGTGTTGGTCATGGA |
| Has-circ-0001756 (Convergent primer) | Forward | AAGGTCATCGACTTTGGTTCA |
| Has-circ-0001756 (Convergent primer) | Reverse | GCACATGTGAGGCCATACCT |
| β2-M (Convergent) | Forward | GAATTGCTATGTGTCTGGGT |
| β2-M (Convergent) | Reverse | CATCTTCAAACCTCCATGATG |
| β2-M (Divergent) | Forward | AGATGAGTATGCCTGCCGTG |
| β2-M (Divergent) | Reverse | TCATCCAATCCAAATGCGGC |

**Table S2**

| **siRNAs** |  |
| --- | --- |
| CircHIPK2 si circ-1 | CCAGAUAUUACAGGUAUGGTT |
| CircHIPK2 si circ-2 | UAUUACAGGUAUGGCCUCATT |
| si PTBP1 | GCGUGAAGAUCCUGUUCAA |
| si nc | UUCUCCGAACGUGUCACGUTT |

**Table S3**

| **mimics and inhibitors** |  |
| --- | --- |
| mimics miR-185-3P | AGGGGCUGGCUUUCCUCUGGUC |
| mimics nc | UUCUCCGAACGUGUCACGUTT |
| anti-miR-185-3P | GACCAGAGGAAAGCCAGCCCCU |
| anti-nc | ACGUGACACGUUCGGAGAATT |

**Table S4**

| **FISH probes** |  |
| --- | --- |
| CircHIPK2 | Biotin-TGTGAGGCCATACCTGTAATATCTGGACTG |
| miR-198 | Digoxin-GACCAGAGGAAAGCCAGCCCCT |

**Table S5**

| **Pull-down probes** |  |
| --- | --- |
| CircHIPK2 Probe-1 | Biotin- CACATGTGAGGCCATACCTGTAATATCTGGACTGCAAGTAG |
| CircHIPK2 Probe-2 | Biotin- GAGGCCATACCTGTAATATCTGGACTGCAAGTAGGTGGAGCA |
| CircHIPK2 Probe-3 | Biotin- CTTGCACATGTGAGGCCATACCTGTAATATCTGG |
| NC Probe | Biotin- CAGTCCAGATATTACAGGTATGGCCTCACA |

**Table S6**

| **Biotinylated probes** |  |
| --- | --- |
| CircHIPK2 | GTGAGGCCATACCTGTAATATCTGGA 3' bio |
| Olige probe | TCCAGATATTACAGGTATGGCCTCAC 3'bio |

**Table S7. Patients and tumor characteristics, hsa_circ_0001756 expression in GC samples**

| **No.** | **Gender** | **Age（yr）** | **TNM** | **Size（diameter,cm）** | **Relative Expression level（ΔCt*）** |
| --- | --- | --- | --- | --- | --- |
| **1** | **M** | **68** | **T3N3bM0** | **6** | **10.21** |
| **2** | **F** | **52** | **T4aN3bM1** | **4.5** | **7.23** |
| **3** | **M** | **69** | **T4aN1M0** | **7** | **11.16** |
| **4** | **F** | **46** | **T4aN1M0** | **6** | **12.32** |
| **5** | **F** | **58** | **T4aN3bM1** | **8.5** | **7.74** |
| **6** | **M** | **68** | **T4aN3M1** | **5.5** | **11.54** |
| **7** | **F** | **59** | **T4aN3bM1** | **5.5** | **9.39** |
| **8** | **F** | **55** | **T4aN3bM1** | **7.5** | **10.41** |
| **9** | **F** | **57** | **T1bN0M0** | **3.5** | **12.53** |
| **10** | **F** | **69** | **T3N1M0** | **3** | **10.23** |
| **11** | **M** | **52** | **T3N1M0** | **7** | **12.32** |
| **12** | **F** | **65** | **T4aN3aM0** | **12** | **11.27** |
| **13** | **M** | **51** | **T3N2M0** | **3** | **11.28** |
| **14** | **M** | **52** | **T1aN0M0** | **1** | **10.97** |
| **15** | **F** | **69** | **T4aN3bM0** | **14** | **7.63** |
| **16** | **M** | **75** | **T3N3aM0** | **6** | **9.01** |
| **17** | **M** | **73** | **T4aN3bM0** | **5.5** | **7.69** |
| **18** | **F** | **49** | **T4aN3M1** | **4** | **8.04** |
| **19** | **F** | **77** | **T2N0M0** | **9.5** | **9.04** |
| **20** | **M** | **52** | **T2N1M0** | **2.5** | **10.96** |
| **21** | **F** | **37** | **T2N0M0** | **14** | **10.02** |
| **22** | **F** | **66** | **T3N1M0** | **6** | **9.67** |
| **23** | **F** | **40** | **T3N1M0** | **6** | **11.5** |
| **24** | **F** | **30** | **T2N0M0** | **2.2** | **8.56** |
| **25** | **M** | **80** | **T4aN1M0** | **6.5** | **12.05** |
| **26** | **F** | **56** | **T2N0M0** | **3** | **11.82** |
| **27** | **F** | **57** | **T3N1M0** | **5.5** | **10.96** |
| **28** | **M** | **64** | **T3N2M1** | **8** | **7.64** |
| **29** | **M** | **74** | **T2N1M0** | **9** | **8.73** |
| **30** | **M** | **66** | **T4N3M0** | **6** | **11.98** |
| **31** | **F** | **69** | **T3N3aM1** | **4** | **9.04** |
| **32** | **M** | **70** | **T2N0M0** | **2** | **11.82** |
| **33** | **M** | **73** | **T4aN3aM1** | **6** | **10.51** |
| **34** | **F** | **68** | **T2N0M0** | **5** | **10.99** |
| **35** | **F** | **66** | **T4aN3aM0** | **3.5** | **8.71** |
| **36** | **M** | **68** | **T4N3M1** | **8** | **11.69** |
| **37** | **M** | **65** | **T3N0M0** | **9** | **7.42** |
| **38** | **F** | **56** | **T2N0M0** | **4.5** | **13.04** |
| **39** | **M** | **54** | **T4aN3aM1** | **7.5** | **11.15** |
| **40** | **F** | **60** | **T3N3aM0** | **2.5** | **11.78** |
| **41** | **M** | **62** | **T4N2M0** | **4** | **6.51** |
| **42** | **F** | **72** | **T2bN1M0** | **2.5** | **11.27** |
| **43** | **M** | **52** | **T1bN1M0** | **2** | **12.64** |
| **44** | **M** | **80** | **T1bN0M0** | **1.5** | **11.313** |
| **45** | **M** | **60** | **T1N0M0** | **2** | **11.83** |
| **46** | **M** | **59** | **T2N1M0** | **4** | **12.89** |
| **47** | **F** | **37** | **T4aN3aM1** | **4** | **10.87** |
| **48** | **F** | **56** | **T4N2M0** | **5.5** | **11.88** |
| **49** | **M** | **54** | **T2N1M0** | **7** | **5.42** |
| **50** | **F** | **64** | **T4aN2M0** | **3.5** | **9.13** |
| **51** | **F** | **52** | **T2N1M0** | **3** | **11.66** |
| **52** | **M** | **66** | **T4aN3M0** | **9** | **9.24** |
| **53** | **M** | **61** | **T4aN0M0** | **7** | **9.3** |
| **54** | **M** | **45** | **T4N2M0** | **10** | **8.32** |
| **55** | **F** | **39** | **T4N3M1** | **4** | **11.67** |
| **56** | **M** | **63** | **T2aN0M0** | **3** | **10.99** |
| **57** | **M** | **75** | **T1bN0M0** | **6.5** | **9.12** |
| **58** | **M** | **61** | **T2N1M0** | **4** | **14.54** |
| **59** | **F** | **64** | **T4aN3aM0** | **4.5** | **7.44** |
| **60** | **F** | **49** | **T4aN0M0** | **9.5** | **9.15** |
| **61** | **M** | **64** | **T3N2M0** | **7.5** | **8.25** |
| **62** | **M** | **50** | **T2N2M0** | **4** | **8.23** |
| **63** | **F** | **38** | **T4N3M0** | **8** | **8.14** |
| **64** | **M** | **62** | **T4N3M1** | **8** | **10.45** |
| **65** | **F** | **69** | **T1N1M0** | **5** | **8.3** |
| **66** | **F** | **43** | **T4N2M0** | **11.5** | **6.49** |
| **67** | **M** | **70** | **T4N3M1** | **7** | **10.13** |
| **68** | **M** | **73** | **T4N2M0** | **6** | **8.05** |
| **69** | **F** | **66** | **T4N2M0** | **3.5** | **8.12** |
| **70** | **M** | **69** | **T2N0M0** | **2** | **10.94** |
| **71** | **F** | **50** | **T4aN3M1** | **14** | **8.83** |
| **72** | **F** | **52** | **T4N2M0** | **8** | **10.07** |
| **73** | **F** | **68** | **T4N3M1** | **6.5** | **12.97** |
| **74** | **M** | **74** | **T2N0M0** | **6** | **9.15** |

***ΔCt=the ct value of hsa_circ_0001756-the ct value of internal control （β_2_-M）**

**The higher ΔCt value implies lower expression.**

**In vitro functional experiments**

Cell proliferation assay

EdU and Cell Counting Kit-8 (CCK-8) assays were used to detect cell proliferation. For the EdU assay, 5000 different transfected GC cells were added to each well of a 96-well plate, followed by drug treatment (RiboBio, Guangzhou, China) and EdU labelling. Apollo staining and DNA staining were performed after fixation with paraformaldehyde, and fluorescence microscopy was performed immediately after completion of staining. For the CCK-8 assay, the treated cells were inoculated into 96-well plates, and 2 × 103 cells were inoculated in each well for 2-4 h. One hundred microlitres of CCK-8 medium was added, and the plates were further incubated for 2 h. The absorbance at a wavelength of 450 nm was measured with a microplate reader. CCK-8 reagent was added every 24 h for incubation and detection, and a cell growth rate curve was drawn.

Scratch healing test

After the 6-well plate was covered with GC cells, a 100-µL tip was used to draw a vertical line, and the plate was washed three times with PBS. Then, 1% serum was added to the medium, the cells were observed under a microscope at 0, 24 and 36 h, and photos were taken.

Migration and invasion analysis

For migration experiments, 40,000 GC cells were added to serum-free medium and plated into Transwell chambers. Subsequently, 500 mL of 30% serum medium was added to the lower chamber, and fixation with paraformaldehyde was performed overnight. For the migration experiment, Matrigel (BD Biosciences, USA) was diluted 1:8, added to the bottom of the chamber, incubated for 2 h and polymerized into a gel. The following steps were the same as above.

Cell cloning experiment

The cloning assay was performed as described previously [18]. Five hundred different transfected GC cells were added to each well of the 6-well plate. After two weeks of culture, paraformaldehyde was used for fixation, and the colony formation rate was determined by imaging.

Analysis of glycolytic function

The manufacturer’s instructions were used to measure glucose consumption and lactic acid production using a glucose determination kit (Sigma‒Aldrich) and a lactic acid determination kit (BioVision, Mountain View, CA, USA).

Flow cytometry to detect apoptosis levels

GC cells were seeded in a 6-well plate at a density of 250,000 cells per well. After corresponding treatment of the cells in the 6-well plate, they were digested with trypsin, pipetted, and then transferred to a centrifuge tube. Centrifuge at 4°C for 3 minutes at 1500 r /min, discard the supernatant after centrifugation, resuspend the cells in PBS, adjust the cell concentration to 1×106 after counting, and centrifuge the cells again with a low temperature centrifuge for 3 minutes at 1500r/min. Remove the supernatant from the cells, add 0.5ml of Binding buffer to each tube, mix and resuspend the cells, then add 5µl each of Fitc-Annexin V and PI dual dyes, incubate at room temperature for 15 minutes in the dark, and perform flow cytometry on the machine.
